# MAMMAL - Molecular Aligned Multi-Modal Architecture and Language for biomedical discovery

**DOI:** 10.1038/s44386-026-00047-4

**Published:** 2026-05-04

**Authors:** Yoel Shoshan, Moshiko Raboh, Michal Ozery-Flato, Vadim Ratner, Alex Golts, Jeffrey K. Weber, Ella Barkan, Simona Rabinovici-Cohen, Sagi Polaczek, Ido Amos, Ben Shapira, Liam Hazan, Matan Ninio, Sivan Ravid, Michael M. Danziger, Yosi Shamay, Sharon Kurant, Joseph A. Morrone, Parthasarathy Suryanarayanan, Michal Rosen-Zvi, Efrat Hexter

**Affiliations:** 1 IBM Research-Israel, IBM Research, Haifa, Israel; 2 IBM TJ Watson Research Center, IBM Research, New York, NY USA; 3 Faculty of Biomedical Engineering, Technion - IIT, Haifa, Israel

**Keywords:** Computational biology and bioinformatics, Drug discovery

## Abstract

Modern AI (Artificial Intelligence) methods offer new opportunities in pharmacology by enabling improved modeling of disease mechanisms and drug action learned from large and heterogeneous biological datasets. A central challenge is developing models that can jointly integrate disparate biomedical modalities. We introduce **MAMMAL** (**M**olecular **A**ligned **M**ulti **M**odal **A**rchitecture and **L**anguage), a foundation model for cross-modal learning, designed to address the challenges associated with drug discovery tasks. MAMMAL was pre-trained on 2 billion samples across protein and antibody sequences, small molecules, and gene expression profiles, and supports classification, regression, and generative tasks on cross-modal inputs. Across eleven benchmarks covering multiple stages of the drug discovery pipeline, MAMMAL achieves state-of-the-art performance on nine tasks and competitive results on two. In an antibody-antigen binding benchmark, fine-tuned MAMMAL prediction scores significantly outperform AlphaFold3 confidence scores, used here as a reference proxy for binding likelihood, in five of seven antigen targets. The MAMMAL framework and pretrained models are publicly available to support open and collaborative research.

## Introduction

Despite major advances in understanding health and disease, many complex biological processes remain poorly understood. Recent progress in artificial intelligence has raised the prospect that longstanding health challenges may prove treatable with AI-based methods^1^ and that the high failure rate of drugs in clinical trials, where approximately 90% of drug candidates fail to achieve regulatory approval^2^, can be improved. Over the past century, the translation of biological discoveries into effective therapies has matured. The drug discovery journey follows a multi-step pipeline that begins with identifying disease-associated proteins, progresses to finding compounds that can effectively target these proteins, and culminates in optimizing drug candidates to meet stringent standards for efficacy and safety. This process is both costly and labor-intensive, requiring extensive laboratory assays that measure drug-target interactions, characterize cellular responses, and validate therapeutic safety and efficacy^3–5^. Therapeutic modalities range from small molecules, which are stable, easy to manufacture, and suitable for oral delivery^6^, to biologic therapeutics, such as engineered antibodies, which offer high specificity but require more complex manufacturing and delivery^7^.

Accelerating drug discovery and improving the predictive accuracy of its steps have become a central focus in biomedical research, with the goal of streamlining target identification, drug design, and testing^8–10^. Analyzing gene expression profiles, particularly from single-cell RNA sequencing (scRNA-seq), has emerged as a key approach for distinguishing disease-associated cell populations and characterizing drug-induced cellular responses^11–14^. These analyses support target identification, mechanistic understanding, and assessment of drug effects across diverse cellular contexts^15,16^. In parallel, generative models have been increasingly applied to drug design, enabling the synthesis of novel candidate molecules for further evaluation^17–19^. As high-throughput screening assays for measuring drug binding affinity are costly and challenging to scale, accurate prediction of drug-target interactions can significantly enhance drug design, improving both efficacy and precision. Overall, predictive modeling of binding affinity, toxicity, and efficacy in the early stages of the pipeline can reduce reliance on expensive late-stage testing, ultimately saving time and resources in drug development.

A central challenge in developing predictive and generative AI models for drug discovery is determining how the different biomedical modalities and entities should be represented and combined as model inputs and outputs^20^. This challenge is particularly acute for interaction-centric tasks, such as predicting whether a small-molecule drug will bind a specific protein target. While sequence-based representations of molecules (e.g., SMILES) and proteins have demonstrated considerable success, the representation of transcriptomic data as sequences has only recently been explored, leading to a range of emerging approaches^21^.

In this work, we introduce a cross-domain foundation model that represents small molecules, proteins, and transcriptomic data within a unified sequence framework. We present MAMMAL (Molecular Aligned Multi-Modal Architecture and Language), designed to address core challenges in AI-driven drug discovery. Our key contributions are: (1) a novel multi-alignment framework that integrates molecular, protein, and gene-expression inputs using a flexible, structured prompt syntax, enabling modeling of complex multi-entity interactions such as drug-target binding; (2) an architecture tailored to drug discovery that natively incorporates numerical values into its embedding space via continuous projections. This improves precision for tasks such as affinity prediction and structural integration, while supporting both predictive and generative objectives; and (3) a large-scale pre-trained model trained on over 2 billion samples across denoising, infilling, and binding-related tasks. Across 11 benchmarks spanning multiple stages of the drug discovery pipeline, fine-tuned MAMMAL achieves state-of-the-art performance on nine tasks. The model and pre-trained weights are publicly available on https://huggingface.co/ibm/biomed.omics.bl.sm.ma-ted-458m under the name ibm/biomed.omics.bl.sm.ma-ted-458m, providing a platform for advancing research in multimodal biomedical modeling.

## Results

**Fig. 1:**
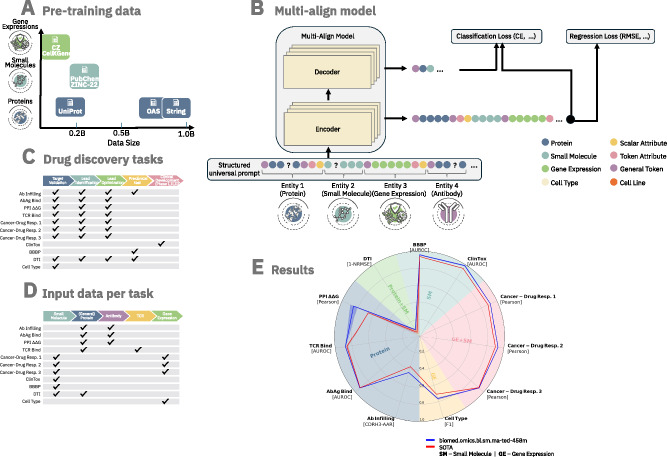
Overview of MAMMAL pretraining data, model architecture, and downstream tasks. **A** We introduce a multi-align model pretrained on six datasets, each containing tens to hundreds of millions of data points. These data points include protein sequences, small molecules, and gene expression profiles, with a combined sample size of 2 billion. **B** The multi-align model combines flexible encoder-only and encoder-decoder components. It takes sequences as input, which may contain any combination of tokens and scalar elements, processed by an encoder stack consisting of self-attention blocks. In encoder-only mode, a dedicated token prediction head outputs logits for token predictions, with an optional scalar prediction head for scalar outputs. In encoder-decoder mode, residual connections inject features from the encoder’s final hidden layer into each decoder layer, and a decoder-specific prediction head outputs the final logits. **C** Diverse downstream tasks performed by the multi-align model, mapped to their contributions within the steps of a typical drug discovery pipeline. **D** Diverse downstream tasks performed by the multi-align model, categorized by data type used in the fine-tuning process. **E** Performance of the multi-align model across a diverse set of tasks compared to SOTA. Panel (**E**) was generated using Matplotlib. Panels (**A**–**D**) were created using Illustrator and PowerPoint.

To evaluate the performance and generalization capabilities of ibm/biomed.omics.bl.sm.ma-ted-458m, we selected a diverse set of existing benchmarks spanning multiple task types and stages of the drug discovery pipeline, prioritizing benchmarks with clearly defined splits when those were available. We assessed model quality through a fine-tuning-based evaluation strategy, where the pretrained model is adapted to each benchmark and compared against specialized state-of-the-art (SOTA) models. The evaluation methodology and fine-tuning protocol as well as detailed descriptions of each benchmark—including background, significance for drug discovery, prior models, and data statistics—are provided in the subsections below. A summary of performance across tasks is presented in Table 1 and visualized in Fig. 1E, and representative encoder-decoder examples are provided in the Supplementary Table S1.

**Table 1:** Comparison of SOTA and MAMMAL Performance Across Benchmarks

Benchmark	Domain	Type	Metric	SOTA	MAMMAL	Imp.
Cell type	GE	cls	*↑* F1	^27^ 0.710	0.763 ± 0.012	**7.5** %
BBBP	SM	cls	*↑* AUROC	^32^ 0.937	0.957 ± 0.006	**2.2** %
ClinTox	SM	cls	*↑* AUROC	^32^ 0.948	0.986 ± 0.007	**4.0** %
Cancer-Drug Response 1	GE+SM	reg	*↑* Pearson	^92^ 0.887	0.917 ± 0.001	**3.4** %
Cancer-Drug Response 2	GE+SM	reg	*↑* Pearson	^92^ 0.900	0.931 ± 0.002	**3.4** %
Cancer-Drug Response 3	GE+SM	reg	*↑* Pearson	^36^ 0.923 [0.917–0.929]	0.928 ± 0.000	0.5 %
Ab Infilling	Protein	gen	*↑* CDRH3-AAR	^47^ 0.375	0.446 ± 0.002	**19.0** %
AbAg Bind	Protein	cls	*↑* AUROC	^63^ 0.924 [0.923–0.925]	0.928 ± 0.002	0.4 %
TCR Bind	Protein	cls	*↑* AUROC	^54^ 0.862 [0.85–0.868]	0.879 ± 0.003	**2.0** %
PPI ΔΔ*G*	Protein	reg	*↑* Pearson	^104^ 0.663	0.852 ± 0.041	**28.5** %
DTI	Prot.+SM	reg	*↓* NRMSE	^60^ 0.942 ± 0.028	0.906 ± 0.011	**3.8**%

For NRMSE, lower is better. For other metrics (AUROC, CDRH3-AAR, Pearson, Spearman, and F1), higher is better. Each row shows the results from a MAMMAL model fine-tuned from ibm/biomed.omics.bl.sm.ma-ted-458m for the corresponding task. In the “Type” column: *cls* classification, *reg* regression, *gen* generation, *Imp.* improvement (percentage) of our model over SOTA. In the “Domain” column: *GE* genes expression, *SM* small molecule, *Prot.* protein.

AlphaFold^22^, whose development contributed to the 2024 Nobel Prize in Chemistry, revolutionized protein structure prediction. Its extension AlphaFold-Multimer^23^ enabled modeling of antibody-antigen complexes, while AlphaFold 3 (AF3)^24^ further improved accuracy and added nucleic acid/small molecule support. Motivated by AF3’s reported advances, we evaluated its performance on therapeutic Antibody and Nanobody complexes (Subsection 2.10). Comparative analysis reveals that MAMMAL achieves better classification performance than AF3 in five of seven targets (Table 2).

**Fig. 2:**
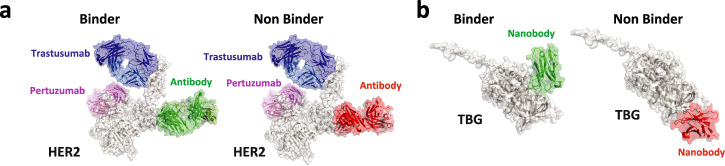
AF3-predicted nanobody binding poses on HER2 and TBG. **a** HER2 extracellular domain (ECD) structure with representative AF3-predicted complexes for a binder and a non-binder. The FDA-approved therapeutic antibodies trastuzumab (blue) and pertuzumab (purple) are shown for reference. AF3 predicts both binders and non-binders engaging the same region of the HER2 ECD, which is distinct from the known therapeutic epitopes, consistent with its poor discriminative performance on this target (AUROC = 0.45). **b** Thyroxine-binding globulin (TBG) structure with AF3-predicted complexes for binding and non-binding VHHs. In contrast to HER2, AF3 predicts distinct binding poses for binders versus non-binders on TBG, consistent with its strong discriminative performance on this target. Visualizations were generated using PyMOL.

### Evaluation

We compiled a comprehensive set of 11 benchmarks covering multiple data domains and task types, including classification, regression and generation, as well as single-entity, multi-entity, and multi-domain tasks. These benchmarks address key stages of the drug discovery process: Identifying target cell types (Cell Type) and advancing precision medicine (Cancer-Drug Response 1-3); predicting drug efficacy (BBBP) and safety (ClinTox); predicting the binding affinity of small-molecule drugs to target proteins (DTI); predicting interactions of biological drugs (PPI); and designing new drugs, such as antibodies, to target specific proteins (Ab Infilling).

To enable fair and direct comparison to prior work, benchmark selection prioritized datasets with predefined train, validation, and test splits or with established splitting strategies reported in the corresponding state of the art studies. For each benchmark, we followed the data splits and evaluation metrics used in the original benchmark or SOTA reference. When explicit train—validation—test splits were available, ibm/biomed.omics.bl.sm.ma-ted-458m was fine tuned on the training set, the best checkpoint was selected using the validation set, and final performance was reported on the test set. For benchmarks evaluated using cross validation, we adopted the same protocol as the corresponding prior work. Unless otherwise noted, standard errors were estimated by training the models with three different random seeds and calculating the standard deviation of their performance on the held out test set. Detailed descriptions of each benchmark, the fine tuning procedures, and the evaluation protocols are provided alongside the corresponding results. For the DTI benchmark, performance is reported using normalized root mean square error (NRMSE), defined as the root mean square error divided by the standard deviation of the test labels. The same normalization is applied to both MAMMAL and reported SOTA results, yielding values below 1 and enabling joint visualization alongside other performance metrics in Fig. 1(E). We consider MAMMAL to outperform existing state of the art when the relative improvement, computed as ∣SOTA − MAMMAL∣/SOTA, exceeds 1%.

### Cell Type Annotation

Cell type prediction enables researchers to distinguish between different cell populations, such as those associated with various diseases^11–14^. It is also crucial for understanding how diseases or drugs affect different cell types. In recent years, a variety of methods have been developed for this task, including approaches based on marker genes, correlation-based techniques, and annotation using classification^25^. Recent advances in transformer-based and large-scale foundation models^26–28^ have shown improved performance.

The input for this task is single-cell gene expression data. The benchmark we used was based on the Zheng68k dataset^29^, which is composed of human peripheral blood mononuclear cells and is widely used for evaluating cell-type annotation performance, due to the similarity of the cell types involved. The dataset contains 68,579 cells across 11 cell types and originally included 32,738 genes, which after removing non-expressed genes leaves 20,387 genes in the benchmark. Preprocessing involved normalization, log transformation of expression values, followed by binning. Similar to the approach in^30^, our model uses a ranked list of expressed gene names, ordered by their expression levels, as input. The label to predict is provided in the cell ontology format “CL:NNNNNN” (see Supplementary Table S1).

Following prior work^27^, we adopted a 5-fold cross-validation strategy to fine-tune and evaluate ibm/biomed.omics.bl.sm.ma-ted-458m, ensuring similar proportions of cell types across folds, and assessed performance using accuracy and macro F1 score. MAMMAL outperforms the previous state-of-the-art performance in both accuracy and F1 (Table 1 and detailed results in Supplementary Table S2), achieving a 7.5% improvement in F1.

### BBBP and ClinTox

To ensure the development of safe and effective drugs, candidates must satisfy rigorous criteria related to both efficacy and safety. In this study, we selected two relevant benchmarks from MoleculeNet^31^, a widely used suite of benchmarks for evaluating machine learning models on small-molecule drug properties: BBBP and ClinTox. The BBBP benchmark focuses on predicting the ability of drugs to penetrate the blood-brain barrier, a critical consideration for drugs targeting the central nervous system. The ClinTox benchmark comprises two related tasks: (1) predicting failure in clinical toxicity trials, and (2) predicting FDA approval status. The overall performance on ClinTox is reported as the average performance across these two tasks.

MoLFormer^32^, a well-established model for molecular embeddings trained on 1.1 billion SMILES sequences, has achieved state-of-the-art performance on both the BBBP and ClinTox benchmarks. In our study, we adopted the benchmarks from^32^, which provided predefined splits for training, validation, and testing. MAMMAL surpasses MoLFormer on both benchmarks (Table 1), achieving an average area under the receiver operating characteristic curve (AUROC) score of 0.957 on BBBP and 0.986 on ClinTox, representing improvements of 2.2% and 4%, respectively, over the state of the art.

### Cancer-Drug Response

Identifying drug response at the cellular level is a critical step in the development of new drugs. Two key public databases supporting this effort, particularly in cancer drug development, are the Cancer Cell Line Encyclopedia (CCLE)^33^ and the Genomics of Drug Sensitivity in Cancer (GDSC)^34^. CCLE provides multi-omics profiles for around 1000 cancer cell lines, while GDSC offers data on the drug responses of these lines to hundreds of drugs, commonly measured using the half-maximal inhibitory concentration (IC_50_). Notable computational models addressed this task^35–37^.

For our study, we used three subsets of the GDSC database: GDSC1 and GDSC2, available through the Therapeutics Data Commons (TDC)^38^, and referred to in the paper as Cancer-Drug Response 1 and Cancer-Drug Response 2, respectively; and a subset published in^36^, referred to as Cancer-Drug Response 3. A dataset statistics table summarizing the number of cell lines, drugs, and cell–drug pairs is provided in Supplementary Table S3. We used the random splits provided by TDC for Cancer-Drug Response 1 and 2, while for Cancer-Drug Response 3, we followed the split methodology outlined in^36^, reserving 5% of the data for the test set, stratified by TCGA^39^ pathways associated with the cancer cell lines.

During fine-tuning, we used only gene-expression profiles and SMILES representations of drugs, as shown in the example prompt in the Supplementary Table S1. Similar to the input format for cell type annotation, gene-expression profiles were provided as ranked lists of gene names based on their expression levels. For predicting continuous IC_50_ values, MAMMAL was utilized in regression mode, taking advantage of its built-in support for floating-point scalar predictions. Our model outperforms the current SOTA models for Cancer-Drug Response 1 and 2 (Table 1), achieving a 3.4% increase in Pearson correlation values. Additionally, it yields results comparable to the SOTA for the Cancer-Drug Response 3 benchmark, with a slight improvement of 0.5%.

To further evaluate MAMMAL’s predictive capability on novel compounds, we assessed drug response predictions for four drugs not present in the GDSC training data: Carfilzomib, Nintedanib, Infigratinib, and Vemurafenib. Tanimoto similarity analysis confirmed that three of these drugs (Carfilzomib, Nintedanib, and Infigratinib) have no structurally similar compounds in the training set (Tanimoto coefficient <0.7), while Vemurafenib shares moderate similarity (0.82) with PLX-4720, a BRAF inhibitor present in GDSC. We performed experimental validation using the same assay protocol employed in GDSC: cell viability was measured using CellTiter-Glo following 72-hour drug incubation, and IC50 values were determined with Prism (GraphPad). The experimental measurements revealed a consistent potency ranking across all tested cell lines: Carfilzomib (most potent), followed by Nintedanib, Infigratinib, and Vemurafenib (least potent). MAMMAL predictions reproduced this exact ranking for the tested cell lines. When extended to all 805 cell lines in GDSC, the model preserved this relative ordering in approximately 90–95% of cases, suggesting that the predicted potency differences are largely cell line–independent.

Notably, Carfilzomib is a proteasome inhibitor approved exclusively for hematological malignancies (multiple myeloma), with limited efficacy in cells of solid tumors^40^. The model’s prediction of Carfilzomib as the most potent agent across diverse solid tumor cell lines aligns with our experimental observations and suggests potential broader applicability that warrants further investigation.

### Ab Infilling

Antibodies are a family of proteins produced by the immune system to neutralize foreign antigens and are of particular interest due to their high specificity and strong binding to target molecules^41,42^. These characteristics have made them a crucial class of therapeutics, driving significant research efforts into the design of new antibody-based drug candidates^7,43–45^. Antigen-binding fragments (Fabs) are the antibody fragments that bind to antigens. It is composed of one constant domain and one variable domain of each of the *heavy* and *light* chains. Each variable region is further divided into four framework (FR) regions and three complementarity-determining regions (CDRs). While FR regions are typically conserved, CDRs exhibit significant variation in their amino acid composition and are generally the primary determinants of binding affinity to the target antigen. When designing novel antibodies for a specific antigen, the typical approach is to explore alternative CDRs that could produce a new, functional antibody with high binding affinity to the target^41,42,46,47^.

Recently, several deep learning methods have been developed for targeted antibody design, framing CDR prediction as an *infilling* task^46–52^. These models predict missing CDR regions, represented by *MASK* tokens, using the amino acid sequences of both the antigen and the antibody’s FR regions. While prior approaches often relied on structural data, this information is scarce and challenging to obtain^53^. In contrast, we fine-tune MAMMAL for the targeted antibody design task using only the sequence data of the antigen and the sequence of the antibody’s FR regions.

The targeted antibody design task benchmark is based on the SAbDab dataset^53^. Following the data processing outlined in^47^, we filtered out samples with missing CDRs to enable direct comparison, even though MAMMAL supports samples that contain missing CDRs. Consistent with^47^, we randomly partitioned the dataset into training, validation, and test folds while ensuring that samples with similar heavy-chain third CDR (CDRH3) sub-sequences remained in the same fold. MAMMAL demonstrates superior amino acid recovery (AAR), defined as the fraction of correctly predicted residues, across all masked CDRs (Table 1; detailed results are provided in Supplementary Table S4). Notably, in CDRH3, the most variable region, it exhibits a remarkable improvement of 19%.

### T-Cell Receptor-Epitope Binding

T-cell receptor (TCR) binding to immunogenic peptides (epitopes) presented by major histocompatibility complex molecules is a critical mechanism in the adaptive immune system, essential for antigen recognition and triggering immune responses. The TCR repertoire exhibits considerable diversity, consisting of an *α*-chain and a *β*-chain that function together to enable T cells to recognize a wide array of epitopes. The *β*-chain is especially significant, as it is crucial for the early stages of T-cell development and possesses greater variability, which enhances the TCR’s capacity to identify diverse pathogens effectively. However, understanding the specific interactions between TCRs and epitopes remains a significant challenge due to the vast variability in TCR sequences. Accurate prediction of TCR-peptide binding from sequence data would advance immunology by offering deeper insights into a patient’s immune status and disease history. This capability holds potential applications in personalized immunotherapy, early diagnosis, and the treatment of diseases such as cancer and autoimmune disorders. In silico tools designed to model TCR-peptide interactions could facilitate the study of therapeutic T-cell efficacy and assess cross-reactivity risks, presenting an opportunity for precision medicine.

We evaluated the model on the task of predicting TCR-epitope binding from sequence data using the Weber benchmark (ref. ^54^, https://tdcommons.ai/multi_pred_tasks/tcrepitope), which consists of 47,182 TCR *β*-chain epitope pairs. This dataset covers 192 distinct epitopes and includes 23,139 unique TCR *β*-chain sequences, with 50% of the pairs serving as negative samples created by randomly pairing TCR sequences with epitopes they are not known to bind with. The dataset also includes the CDR3 subsequence for each TCR *β*-chain, the most hypervariable region of the chain. We used 10-fold cross-validation. The folds were pre-defined in ^54^. Fine-tuning involved three concurrent tasks: TCR *β*-chain mask infilling and two classification tasks: (i) TCR *β*-chain epitope binding prediction and (ii) TCR *β*-chain -CDR3 epitope binding prediction. Here, we report the performance only for the TCR *β*-chain epitope binding prediction task. Our model achieves an average AUROC of 0.879 (Table 1), representing a statistically significant improvement of 2% over the SOTA, as our result falls outside the SOTA’s confidence interval.

### Protein-Protein Interaction - ΔΔG Prediction

An important factor in drug design is binding affinity, commonly measured by the equilibrium dissociation constant, *K*_*D*_, which is related to the Gibbs free energy Δ*G* through the equation

1 $$\Delta G=kT\,{ln}({K}_{D}),$$

where *k* is the Boltzmann constant and *T* is the temperature^55^.

The effect of introducing mutations into a protein–protein complex is commonly quantified by the change in binding free energy relative to the reference (wild-type) complex. This mutation-induced effect is captured by the difference in Gibbs free energy, defined as

$$\Delta \Delta G=\Delta {G}_{{\rm{mutant}}}-\Delta {G}_{{\rm{wild}}-{\rm{type}}}.$$

By subtracting the wild-type free energy, ΔΔ*G* isolates the energetic contribution of the mutation itself. As a result, ΔΔ*G* provides a direct measure of whether a mutation stabilizes or destabilizes binding and is a standard target in studies of mutational effects on protein–protein interactions^56–58^.

The SKEMPI dataset^55^ provides experimentally measured changes in thermodynamic parameters, including Δ*G* and kinetic rate constants, for mutations in protein–protein complexes with known structures in the Protein Data Bank^59^. This dataset is widely used to benchmark methods for predicting mutation-induced changes in binding affinity, particularly ΔΔ*G*. A commonly used subset of SKEMPI comprising 1131 single-point mutations (S1131) is adopted as our benchmark. Following standard practice, we report 10-fold cross-validation performance on this subset. The input for our model consists solely of amino acid sequences for the wild-type and mutant complexes, without structural information. Leveraging MAMMAL–s support for continuous-valued outputs, we formulate ΔΔ*G* prediction as a regression task. Performance results are reported in Table 1. Our model achieves an average Pearson correlation of 0.852, substantially exceeding the previous sequence-only state of the art (0.663), and remains competitive with structure-based methods, falling only 1.6% short of the reported best performance of 0.866^56^.

### Drug-Target Interaction

Predicting drug-target binding affinity plays a crucial role in the early stages of drug discovery. Traditionally, binding affinities are measured through high-throughput screening experiments, which, while accurate, are resource-intensive and limited in their scalability to evaluate large sets of drug candidates. In this task, we focus on predicting binding affinities using p*K*_*D*_, the negative logarithm of the dissociation constant, which reflects the strength of the interaction between a small molecule (drug) and a protein (target). We utilize the PEER (Protein sEquence undERstanding) benchmark^60^ for DTI prediction. This benchmark leverages data from the BindingDB dataset^61^, with a specific test split that holds out four protein classes - estrogen receptor, G-protein-coupled receptors, ion channels, and receptor tyrosine kinases - for assessing generalization performance on unseen classes.

For model fine-tuning, we conducted hyperparameter optimization, selecting an initial learning rate of 0.0004, with no dropout and no weight decay. We standardized the p*K*_*D*_ values based on the mean and standard deviation of the training set. For evaluation, we transformed the predicted values back to their original scale. Our model achieves an average NRMSE of 0.906 (Table 1), demonstrating a solid improvement of 3.8% over the SOTA reported by^60^.

### Antibody-Antigen Binding Prediction

Accurate prediction of antigen-antibody binding can enhance the design and optimization of therapeutic antibodies, leading to improved efficacy and specificity. We employ the human epidermal growth factor receptor 2 (HER2) dataset^62^ as a benchmark for predicting antibody-antigen binding. HER2 is a key target for certain types of breast and stomach cancers. The dataset includes variations of the clinically approved therapeutic antibody trastuzumab and their corresponding affinities for the HER2 antigen. The dataset comprises 8,935 binding and 25,114 non-binding trastuzumab CDR H3 mutants, each with up to 10 mutations, following de-duplication and the removal of samples labeled as both binding and non-binding.

For the most accurate comparison with the SOTA (refs. ^62,63^), the HER2 dataset was divided into train (70%), validation (15%) and test (15%) sets. For increased robustness, the train set was further divided into 5 folds. The reported results are from the 5 models trained on different train folds, and evaluated on the test set.

Finetuning involved feeding the target antigen sequence as well as the entire heavy-chain variable region as input and predicting binding to the target sequence. Our model achieves an average AUROC of 0.928 (Table 1), slightly surpassing the SOTA, which incorporated structural data, unlike our model.

### Comparison of AlphaFold 3 and MAMMAL in Predicting Antibody-Antigen and Nanobody-Antigen Binding

Accurate prediction of antibody-antigen and nanobody-antigen interactions is essential for evaluating therapeutic efficacy and guiding protein engineering. Although AlphaFold 3 (AF3) is not explicitly designed as a binary protein-protein interaction (PPI) classifier, binding likelihood can be inferred from structure-derived confidence scores, such as predicted template modeling (pTM) and interface predicted template modeling (ipTM), computed from predicted protein-protein complexes. These confidence scores are derived from structural predictions rather than classification objectives. Recent studies suggest that these scores correlate with true binding events^64,65^.

Accordingly, we conduct an exploratory comparison between AF3-derived confidence scores and a fine-tuned MAMMAL model for distinguishing binders from non-binders. We emphasize that AF3 provides detailed 3D structural hypotheses, whereas MAMMAL is a sequence-only model that produces probabilistic binding predictions; the comparison is intended to assess relative discriminative power for binding prediction rather than to equate the underlying modeling approaches.

We first evaluated the extracellular domain (ECD) of HER2, a well-characterized therapeutic antigen with experimentally validated binding epitopes. We used the HER2-specific MAMMAL model described in Subsection 2.9. Due to the computational demands and limited availability of AF3, the HER2 benchmark test set was downsampled to 60 examples, comprising 30 binders and 30 non-binders. The HER2-specific MAMMAL model demonstrates strong discriminative performance, achieving an AUROC of 0.88. In contrast, AF3 exhibits no meaningful separation between binders and non-binders (AUROC = 0.45), and the difference in performance between the two models is highly significant (DeLong test, *P* = 1.5 × 10^−6^). Structural analysis further reveals that AF3-predicted binding sites are indistinguishable between binders and non-binders and deviate from the known epitopes of the FDA-approved antibodies trastuzumab and pertuzumab (Fig. 2a). An extended comparative analysis of MAMMAL (pre-trained and fine-tuned) and AF3 is provided in Supplementary Table S5. This includes several AF3 confidence-score variants, using ipTM - and pTM-based scoring, and heavy-chain-only and heavy+light-chain input configurations.

Next, we evaluated nanobody binding across six structurally diverse antigen targets: albumin, mannose receptor (CD206), epidermal growth factor receptor (EGFR), thyroxine-binding globulin (TBG), tumor necrosis factor alpha (TNF*α*), and von Willebrand factor (VWF). Binding nanobodies were collected from SAbDab-nano^66^, patents, and proprietary datasets. Non-binders consisted of nanobodies experimentally confirmed as non-binding in phage-display library screenings, as well as nanobodies targeting unrelated antigens. From a total of 668 nanobody–antigen pairs (131 binders and 537 non-binders), we selected 475 sequences (64 binders and 411 non-binders) for MAMMAL fine-tuning and reserved 193 sequences (67 binders and 126 non-binders) for held-out evaluation. A single MAMMAL model was fine-tuned on training data comprising binders and non-binders across all targets, and subsequently evaluated separately on the test subset corresponding to each target, with performance compared against AF3 confidence scores computed on the same samples. Test set statistics and per-target MAMMAL and AF3 performance are summarized in Table 2. Additional performance metrics for MAMMAL and AF3 are presented in Supplementary Tables S6–S7. As shown, MAMMAL significantly outperforms AF3 on the larger targets: albumin, CD206, EGFR, and VWF. In contrast, AF3 achieves superior performance on the smaller TBG target, and analysis of the predicted structures highlights distinct binding sites for binders versus non-binders (Fig. 2b and Supplementary Figure S4). For the smallest protein, TNF*α*, the two models exhibit comparable performance.

**Table 2:** Per-target AUROC comparison between MAMMAL and AF3 on held-out antibody/nanobody–antigen test subsets

Target	length (AA)	*n*	pos	MAMMAL AUROC	AF3 AUROC	ΔAUROC	*P*-value
HER2 ECD	630	60	30	**0.93**	0.45	**+0.42**	1.5 × 10^−6^
Albumin	609	28	11	**0.91**	0.59	**+0.32**	7.4 × 10^−3^
CD206	1,456	37	14	**1.00**	0.59	**+0.41**	1.1 × 10^−5^
EGFR	1,210	28	11	**0.94**	0.49	**+0.45**	2.2 × 10^−5^
TBG	384	32	5	0.63	**1.00**	−0.37	1.0 × 10^−4^
TNF*α*	233	34	13	0.86	**0.87**	−0.01	NS
VWF	2,813	34	13	**0.83**	0.32	**+0.51**	3.9 × 10^−6^

*P*-values were computed using the DeLong test for correlated ROC curves. ΔAUROC denotes AUROC_MAMMAL_ − AUROC_AF3_. NS indicates a non-significant difference (*p* > 0.1). ECD denotes the extracellular domain. Bold in the AUROC column of a method (MAMMAL or AF3) indicates higher result for that method. Bold in the ΔAUROC column indicates rows where MAMMAL outperforms AF3.

## Discussion

Recent progress in biomedical foundation models has enabled substantial yet largely domain-specific advances across cellular and molecular biology. Specialized model classes-including RNA expression-based models^26,28,30^, protein language models^67,68^, and frameworks for small-molecule-protein interaction modeling^32^-provide increasingly detailed representations of biological systems. Together, these efforts suggest the feasibility of integrative frameworks such as the proposed AI-enabled “virtual cell”^69,70^. Realizing this vision, however, requires approaches that can coherently align heterogeneous biological modalities within a scalable and unified representation.

MAMMAL advances this goal by reframing diverse biological tasks as sequence-to-sequence problems and introducing design choices tailored to drug discovery applications. Its multi-domain syntax and extensible tokenizer allow heterogeneous biological inputs and outputs to be expressed within a shared sequence representation. The implementation of continuous embeddings for numerical variables preserves quantitative precision without the information loss typical of discretization, facilitating integration of biochemical and pharmacological measurements. MAMMAL further supports both encoder-only and encoder-decoder configurations, offering the architectural flexibility required to address the broad spectrum of predictive and generative tasks encountered throughout the drug discovery pipeline. The strong performance of fine-tuned models across multiple domains and stages highlights the practical utility of this unified framework in established in silico benchmarks.

In addition to benchmark evaluations, MAMMAL was assessed in real-world settings, where it accurately predicted the relative potency ranking of four drugs, including Carfilzomib^40^, a proteasome inhibitor approved only for hematological malignancies, across solid tumor cell lines. Wet-lab validation using the same CellTiter-Glo assay protocol as GDSC confirmed the predicted ordering. This result demonstrates the model’s capacity to generalize to novel compounds and identify potentially repurposable agents for indications beyond their current approval. In parallel, MAMMAL has been employed in joint studies with academic and industry collaborators, including a published investigation supporting its ability to predict antibody activity against influenza hemagglutinin proteins^71^. Additional collaborative studies involving prospective wet-lab validation of MAMMAL-guided predictions are currently in progress, further evaluating the framework across diverse biological and therapeutic contexts.

We further evaluated MAMMAL for antibody binder versus non-binder identification and compared it to AlphaFold 3 (AF3), which has recently been explored as a proxy for estimating binding propensity via predicted interfaces^64,65^. Fine-tuned MAMMAL achieved robust discriminative performance across six of seven antigens-including flexible, glycosylated, and multi-domain targets such as CD206, EGFR, HER2, albumin, and VWF-with AUROCs ranging from 0.83 to 1.00. In contrast, AF3, applied in a zero-shot setting, showed limited ability to separate binders from non-binders for most targets (AUROC ≤ 0.59), performing well only on the rigid globular antigen TBG and matching MAMMAL on TNF*α*. These limitations may reflect AF3’s bias toward true-positive PDB complexes, its lack of explicit negative supervision, and its reliance on a single static conformation. As a result, AF3 struggles with proteins containing heterogeneous or intrinsically disordered regions (IDRs), which comprise 30–40% of the human proteome and are known to challenge structure-based models^72^, including IDR-rich therapeutic targets such as EGFR^73^ and HER2^74^. In contrast, protein language models (PLMs) such as MAMMAL better capture the statistical properties of IDRs, consistent with observations that pLM-based approaches perform well on IDR-related benchmarks^75^. These factors together likely contribute to MAMMAL’s improved robustness for binder identification across therapeutically relevant targets.

Text-based large language models excel at understanding and reasoning over human language and have been adapted to the biomedical domain through pretraining on PubMed abstracts and full-text articles. However, these models operate primarily on unstructured text and do not explicitly represent structured biological modalities. In a comparison with BioMedLM^76^, reported in the Supplementary Information, MAMMAL outperformed text-only models on tasks involving proteins and gene expression data, highlighting the limitations of language-only pretraining for biological data lying outside natural language, such as protein sequences and gene expression measurements. Beyond text-based LLMs, prior work includes graph neural networks, which encode molecular, protein, and biological-system topology and can directly represent knowledge graphs^77^. While sequence-based representations can implicitly capture aspects of three-dimensional structure^78^, explicitly modeling 3D information can further improve performance, motivating sequence models that input or generate structural representations^79–81^. More recently, diffusion models have emerged as powerful methods for modeling molecules and proteins directly in continuous atomic coordinate space^24,82–84^. In contrast to these architecture- or modality-specific approaches, MAMMAL adopts a unifying sequence-to-sequence abstraction that decouples task definition from architectural design. A key open challenge is how to combine this simplicity and flexibility with the expressive power of specialized models, particularly diffusion-based architectures that excel at high-fidelity structural generation.

The growing adoption of agentic workflows-leveraging LLMs and specialized agents to automate and accelerate discovery processes-marks a broader shift in biomedical research practice^85,86^. Fine-tuned MAMMAL models integrate naturally into this paradigm, providing modular, task-specific components that support scalable and efficient discovery workflows. Fair and meaningful comparisons across biomedical models require benchmarks with accessible datasets, reproducible data splits, and clear evaluation protocols. Although the benchmarks used here satisfy these criteria and span multiple stages of the drug discovery pipeline, our evaluation is limited to models that publicly report results on these tasks, and claims of state-of-the-art performance should therefore be interpreted within this scope. Continued development of open benchmarks and models is essential for advancing robust and generalizable approaches for drug discovery. We invite the community to evaluate their own models under this benchmark suite and to explore MAMMAL on new datasets and tasks.

## Methods

The MAMMAL method is built around three core components: The model architecture, the molecular prompt syntax, and extensive pretraining. In Subsection 4.1, we detail the architecture and its enhancements to the standard transformer.

Subsections 4.2 and 4.3 focus on the molecular representations and prompt syntax, key features that enable the support of a diverse range of pretraining and downstream tasks for drug discovery. Finally, Subsection 4.4 outlines the pretraining process, which facilitates leveraging large, cross-domain datasets and handling multiple entities simultaneously.

### MAMMAL Architecture

The MAMMAL architecture builds upon the transformer architecture introduced by Vaswani et al.^87^ and draws inspiration from the T5 framework^88^ while introducing several key modifications on top of it. This design conceptualizes tasks as sequence-to-sequence problems within a unified model, introducing three key features:

- **Optimized for Representation and Generation Tasks:** Biomedical models for drug discovery must perform diverse tasks, ranging from token-level or prompt-level representation to tasks requiring strong generative capabilities. For instance^89^, introduced a non-causal, decoder-only protein language model optimized for both understanding and generating protein-related data, outperforming state-of-the-art baselines. MAMMAL is designed to jointly optimize for such diverse tasks, enabling the model to learn from multiple stages of the drug discovery pipeline. This collective learning is facilitated by a shared encoder, which bridges gaps between distinct AI applications in drug discovery. Specifically, MAMMAL supports both encoder-only and encoder-decoder autoregressive modes. Encoder-only mode excels in representation-heavy tasks, such as classification and regression, while encoder-decoder mode is better suited for generative tasks. By sharing encoder stack weights across these modes, MAMMAL facilitates efficient multi-task training, with parameter updates conducted through gradient accumulation across all tasks.
- **Flexible Multi-Domain Structured Prompts:** Drug discovery tasks often require prompts that describe molecular complexes composed of multiple entities. These prompts may also include attributes related to the entire complex or specific internal entities to improve prediction accuracy. To address this, MAMMAL employs a modular tokenizer that enables multi-domain structured prompts by assigning distinct sub-tokenizers to each entity type. Compared to free-text prompts, structured prompts provide a consistent and explicit representation of input data, capturing relationships and attributes between entities, and facilitating the training. While MAMMAL primarily focuses on structured input, incorporating free text as an additional data source represents a natural extension. Recent advancements in biomedical large language models^90–92^, demonstrate the potential of free-text prompts to enhance versatility and expand training datasets. Such integration could integrate free text directly, mix it with structured prompts, or leverage embeddings derived from extensively pre-trained text models^93^, https://github.com/ibm-granite/granite-3.0-language-models/. These embeddings could harness the latent knowledge encoded within external models to enrich the system’s performance.
- **Numerical Values Integration:** A notable innovation of MAMMAL is its native support for numerical values (e.g., 2, 3.14, 10,000.1) as both inputs and outputs. Numerical data is crucial for modeling drug discovery tasks and allows for direct representation of important attributes. This capability expands the range of tasks MAMMAL can handle, such as binding affinity prediction and protein folding. Many biomedical large language models either lack support for numerical data^90^, employ limited solutions such as binning^92^, or rely on digit-based representations^91^, which can inflate input length^94^, offer compelling digit-based approach of splitting the number into semantic parts (mainly digits), supporting arbitrary scalar values while adding only a limited number of tokens to the vocabulary. While this simplifies integration with standard language models, it inflates the number of input/output tokens significantly. This is especially evident in tasks like gene expression prediction which require supporting thousands of scalars in a single prompt and expected model output^95^, suggests a new loss term that considers numerical proximity, with the main advantage of not requiring any additional prediction head for scalars, allowing to introduce numerical values support within existing language model architectures. However, it is currently limited to a fixed vocabulary, not allowing support for arbitrary (possibly unseen in train time) scalar values. Other methods, such as discretization, demonstrated in^81^ through pre-trained VQ-VAE^96^ that encodes and decodes 3D spatial positions, represent another approach limited to a specific use case. MAMMAL, however, integrates continuous numerical values directly into its embedding space via a projection layer. The resulting embeddings align with the input token embeddings, ensuring simple, efficient, and effective utilization of numerical data.

Further details about the MAMMAL architecture can be found in Supplementary Figure S1.

### Entity Representation

Each entity domain supported by MAMMAL makes use of a representation approach that has been selected as follows:

- **Small Molecules**. Represented using SMILES sequences (Simplified Molecular Input Line Entry System). For example, paracetamol is represented as CC(=O)NC1=CC=C(O)C=C1.
- **Gene Expression**. Represented as an ordered list of gene names, sorted in descending order based on log-normalized and binned expression values. In case of ties, genes are sorted alphabetically. This representation applies to both single-cell and bulk RNA expression data.
- **Proteins**. Represented as concatenated amino acid chains, preserving original source-data order. This representation contains only sequence data, without structural information.
- **Antibodies**. Represented similarly to proteins, using concatenated amino acid sequences. Each chain is prefixed with a token indicating its type (heavy or light).

### Prompt Syntax

The prompt syntax is built around a modular tokenizer architecture that governs how molecular data is interpreted and encoded. At its core, the tokenizer defines a common syntax using special tags that represent molecular entities, sequences, attributes, and interactions across diverse molecular systems. It supports multi-domain inputs by delegating different segments of the input sequence to specialized sub-tokenizers, each responsible for handling a specific data type or modality.

All sub-tokenizers operate under the umbrella of the main tokenizer and share access to a unified set of special tokens, such as 〈EOS〉, ensuring consistency across domains. Among these sub-tokenizers, a designated numeric sub-tokenizer is used for handling continuous values. Instead of tokenizing numeric values into discrete tokens, this sub-tokenizer projects them directly into the model’s embedding space via a learned projection layer.

The prompt syntax applies uniformly to both inputs and outputs of the model. It is also designed to be extensible: New tags can be introduced into individual sub-tokenizers or added to the shared token set without disrupting existing functionality. Thanks to this modular structure, updates to domain-specific sub-tokenizers or the token vocabulary maintain backward compatibility, enabling interoperability between newly trained and previously deployed models. More detailed explanations and examples of the prompt syntax can be found in Supplementary Figures S2, S3 and Supplementary Table S1.

### Pretraining

MAMMAL is designed as a comprehensive foundation model, capable of spanning multiple domains and accommodating a variety of entities. It is intended to support diverse task types, ranging from representation-focused tasks to generation-oriented ones. To achieve this, MAMMAL is trained on multiple tasks concurrently. Pretraining was conducted on two billion samples sourced from six datasets, which are all publicly available, covering three distinct domains across seven tasks. Table 3 summarizes these tasks, detailing the relevant domains, entity types, and specific datasets. Additional details about the pretraining are provided in the Supplementary Information.

**Table 3:** Pretraining Tasks

Name	Domain	Entity Type	Task Type	Dataset	Number of Samples
Protein LM	Biologic	General Protein	Spans Masking LM	Uniref90^67^	180M
Antibody LM	Biologic	Antibody	Spans Masking LM	OAS^98^	650M
Small Molecule LM	Small Molecules	Small Molecule	Spans Masking LM	ZINC^100^ + PubChem^101^	200M
Cell Genes LM	Single Cell Transcript- omics	Cell Genes	Spans Masking LM	CELLxGENE^103^	30M
Protein-Protein Interaction	Biologic	General Protein	Classification	STRING^102^	780M
Protein-Protein Interaction Gen.	Biologic	General protein	Generation	STRING^102^	390M
Antibody Denoise	Biologic	Antibody	Denoise Sequence	OAS^98^	650M

Details on the pretraining tasks that were used while training ibm/biomed.omics.bl.sm.ma-ted-458m. “Number of Samples” lists the post-filtering number of samples actually used. A single model was pretrained with all of the listed tasks, accumulating knowledge spanning multiple domains.

## Supplementary information

Supplementary information

## Data Availability

All datasets used in this study are publicly available. **Cell Type**. The Zheng68k dataset was obtained from https://www.10xgenomics.com/datasets/fresh-68-k-pbm-cs-donor-a-1-standard-1-1-0(file: https://cf.10xgenomics.com/samples/cell-exp/1.1.0/fresh_68k_pbmc_donor_a/fresh_68k_pbmc_donor_a_filtered_gene_bc_matrices.tar.gz) **BBBP and ClinTox** benchmarks were obtained from https://github.com/IBM/molformer/tree/main/datathat points to https://ibm.ent.box.com/v/MoLFormer-data(file: finetune_datasets.zip). **Cancer-Drug Response 1 and 2**. The GDSC1 and GDSC2 benchmarks were accessed with random splits from the TDC library (https://pypi.org/project/PyTDC/). **Cancer-Drug Response 3**. The benchmark was obtained from the DeepCDR^36^ git repository (https://github.com/kimmo1019/DeepCDR/tree/master/data). **DTI**. This benchmark was published by^60^ and is available in https://torchdrug.ai/docs/api/datasets.html#bindingdb**Ab Infilling**. This data was taken from^47^, which provides a preprocessed subset of the publicly available SAbDab database^53^. The preprocessing pipeline includes a similarity-based clustering for the data splits and sample-level filtering that excludes samples considered invalid in^47^. For additional information, we refer to^47^ and the publicly available codebase: https://github.com/THUNLP-MT/dyMEAN. **PPI*****Δ******Δ******G***. The SKEMPI S1131 dataset of non-redundant single mutations was derived from SKEMPI^55^ in^97^ and can be downloaded from https://zhanggroup.org/BindProfX/download/. **TCR Bind** The Weber TCR binding dataset was downloaded from https://tdcommons.ai/multi_pred_tasks/tcrepitope**Antibody-Antigen Bind** The HER2 antibody-antigen binding dataset was taken from the original paper^62^ github repository https://github.com/dahjan/DMS_opt. **Pretraining**. The ibm/biomed.omics.bl.sm.ma-ted-458m was pre-trained over OAS^98^, UniProt^99^, Zinc^100^, PubChem^101^, STRING^102^ and CELLxGENE^103^. Supplementary Information describes the pre-processing steps applied.

## Code availability

The model architecture, fine-tuning framework, and end-to-end examples are publicly available at: https://github.com/BiomedSciAI/biomed-multi-alignment. This GitHub repository provides comprehensive resources for working with the model, including setup instructions, fine-tuning guidelines, and inference workflows for multiple downstream tasks. Pretrained model weights and the associated tokenizer are hosted on the Hugging Face Model Hub and can be accessed at: https://huggingface.co/ibm/biomed.omics.bl.sm.ma-ted-458m. Additionally, selected fine-tuned model checkpoints and tokenizers are available via: https://huggingface.co/models?other=base_model:finetune:ibm-research/biomed.omics.bl.sm.ma-ted-458m, and can be explored interactively through the Hugging Face Space at: https://huggingface.co/spaces/ibm/biomed-multi-alignment.

## References

[1] Cheng, F. et al. Artificial intelligence and open science in discovery of disease-modifying medicines for Alzheimer’s disease. *Cell Rep. Med.* **5**, 101379 (2024). 10.1016/j.xcrm.2023.101379 PMC10897520 38382465

[2] Mullard, A. Parsing clinical success rates. *Nat. Rev. Drug Discov.* **15**, 447–448 (2016). 27357012 10.1038/nrd.2016.136

[3] Paul, S. M. et al. How to improve r&d productivity: the pharmaceutical industry’s grand challenge. *Nat. Rev. Drug Discov.* **9**, 203–214 (2010). 20168317 10.1038/nrd3078

[4] DiMasi, J. A., Grabowski, H. G. & Hansen, R. W. Innovation in the pharmaceutical industry: new estimates of r&d costs. *J. Health Econ.* **47**, 20–33 (2016). 26928437 10.1016/j.jhealeco.2016.01.012

[5] Wouters, O. J., McKee, M. & Luyten, J. Estimated research and development investment needed to bring a new medicine to market, 2009-2018. *Jama* **323**, 844–853 (2020). 32125404 10.1001/jama.2020.1166 PMC7054832

[6] Southey, M. W. & Brunavs, M. Introduction to small molecule drug discovery and preclinical development. *Front. Drug Discov.* **3**, 1314077 (2023).

[7] Lu, R.-M. et al. Development of therapeutic antibodies for the treatment of diseases. *J. Biomed. Sci.* **27**, 1–30 (2020). 31894001 10.1186/s12929-019-0592-z PMC6939334

[8] Sadybekov, A. V. & Katritch, V. Computational approaches streamlining drug discovery. *Nature* **616**, 673–685 (2023). 37100941 10.1038/s41586-023-05905-z

[9] Huang, D., Yang, M., Wen, X., Xia, S. & Yuan, B. Ai-driven drug discovery: Accelerating the development of novel therapeutics in biopharmaceuticals. *J. Knowl. Learn. Sci. Technol. ISSN: 2959-6386 (online)* **3**, 206–224 (2024).

[10] Son, A. et al. Revolutionizing molecular design for innovative therapeutic applications through artificial intelligence. *Molecules* **29**, 4626 (2024). 39407556 10.3390/molecules29194626 PMC11477718

[11] Baslan, T. & Hicks, J. Unravelling biology and shifting paradigms in cancer with single-cell sequencing. *Nat. Rev. Cancer* **17**, 557–569 (2017). 28835719 10.1038/nrc.2017.58

[12] Ofengeim, D., Giagtzoglou, N., Huh, D., Zou, C. & Yuan, J. Single-cell rna sequencing: unraveling the brain one cell at a time. *Trends Mol. Med.* **23**, 563–576 (2017). 28501348 10.1016/j.molmed.2017.04.006 PMC5531055

[13] Rozenblatt-Rosen, O., Stubbington, M. J., Regev, A. & Teichmann, S. A. The human cell atlas: from vision to reality. *Nature* **550**, 451–453 (2017). 29072289 10.1038/550451a

[14] Potter, S. S. Single-cell rna sequencing for the study of development, physiology and disease. *Nat. Rev. Nephrol.* **14**, 479–492 (2018). 29789704 10.1038/s41581-018-0021-7 PMC6070143

[15] Van de Sande, B. et al. Applications of single-cell rna sequencing in drug discovery and development. *Nat. Rev. Drug Discov.* **22**, 496–520 (2023). 37117846 10.1038/s41573-023-00688-4 PMC10141847

[16] Dann, E. et al. Estimating the impact of single-cell rna sequencing of human tissues on drug target validation. *medRxiv* https://www.medrxiv.org/content/early/2024/10/22/2024.04.04.24305313 (2024).

[17] Tang, X. et al. A survey of generative ai for de novo drug design: new frontiers in molecule and protein generation. *Brief. Bioinforma.* **25**, bbae338 (2024). 10.1093/bib/bbae338 PMC11247410 39007594

[18] Shanehsazzadeh, A. et al. Unlocking de novo antibody design with generative artificial intelligence. *bioRxiv* 2023–01 (2023).

[19] Swanson, K. et al. Generative ai for designing and validating easily synthesizable and structurally novel antibiotics. *Nat. Mach. Intell.* **6**, 338–353 (2024).

[20] Athaya, T., Ripan, R. C., Li, X. & Hu, H. Multimodal deep learning approaches for single-cell multi-omics data integration. *Brief. Bioinforma.* **24**, bbad313 (2023). 10.1093/bib/bbad313 PMC10516349 37651607

[21] Szałata, A. et al. Transformers in single-cell omics: a review and new perspectives. *Nat. methods* **21**, 1430–1443 (2024). 39122952 10.1038/s41592-024-02353-z

[22] Jumper, J. et al. Highly accurate protein structure prediction with AlphaFold. *Nature* **596**, 583–589 (2021). 34265844 10.1038/s41586-021-03819-2 PMC8371605

[23] Evans, R. et al. Protein complex prediction with alphafold-multimer. *bioRxiv* https://www.biorxiv.org/content/early/2022/03/10/2021.10.04.463034 (2022).

[24] Abramson, J. et al. Accurate structure prediction of biomolecular interactions with AlphaFold 3. *Nature* **630**, 493–500 (2024). 38718835 10.1038/s41586-024-07487-w PMC11168924

[25] Qi, R., Ma, A., Ma, Q. & Zou, Q. Clustering and classification methods for single-cell rna-sequencing data. *Brief. Bioinforma.* **21**, 1196–1208 (2020). 10.1093/bib/bbz062 PMC7444317 31271412

[26] Cui, H. et al. scgpt: toward building a foundation model for single-cell multi-omics using generative AI. *Nat. Methods* 1–11 (2024). 10.1038/s41592-024-02201-0 38409223

[27] Xu, J., Zhang, A., Liu, F., Chen, L. & Zhang, X. Ciform as a transformer-based model for cell-type annotation of large-scale single-cell rna-seq data. *Brief. Bioinforma.* **24**, bbad195 (2023). 10.1093/bib/bbad195 37200157

[28] Yang, F. et al. scbert as a large-scale pretrained deep language model for cell type annotation of single-cell rna-seq data. *Nat. Mach. Intell.* **4**, 852–866 (2022).

[29] Zheng, G. X. et al. Massively parallel digital transcriptional profiling of single cells. *Nat. Commun.* **8**, 14049 (2017). 28091601 10.1038/ncomms14049 PMC5241818

[30] Theodoris, C. V. et al. Transfer learning enables predictions in network biology. *Nature* **618**, 616–624 (2023). 37258680 10.1038/s41586-023-06139-9 PMC10949956

[31] Wu, Z. et al. Moleculenet: a benchmark for molecular machine learning. *Chem. Sci.* **9**, 513–530 (2018). 29629118 10.1039/c7sc02664a PMC5868307

[32] Ross, J. et al. Large-scale chemical language representations capture molecular structure and properties. *Nat. Mach. Intell.* **4**, 1256–1264 (2022).

[33] Barretina, J. et al. The cancer cell line encyclopedia enables predictive modelling of anticancer drug sensitivity. *Nature* **483**, 603–607 (2012). 22460905 10.1038/nature11003 PMC3320027

[34] Yang, W. et al. Genomics of drug sensitivity in cancer (gdsc): a resource for therapeutic biomarker discovery in cancer cells. *Nucleic acids Res.* **41**, D955–D961 (2012). 23180760 10.1093/nar/gks1111 PMC3531057

[35] Lind, A. P. & Anderson, P. C. Predicting drug activity against cancer cells by random forest models based on minimal genomic information and chemical properties. *PloS one* **14**, e0219774 (2019). 31295321 10.1371/journal.pone.0219774 PMC6622537

[36] Liu, Q., Hu, Z., Jiang, R. & Zhou, M. Deepcdr: a hybrid graph convolutional network for predicting cancer drug response. *Bioinformatics* **36**, i911–i918 (2020). 33381841 10.1093/bioinformatics/btaa822

[37] Liu, X. et al. Graphcdr: a graph neural network method with contrastive learning for cancer drug response prediction. *Brief. Bioinforma.* **23**, bbab457 (2022). 10.1093/bib/bbab457 34727569

[38] Huang, K. et al. Therapeutics Data Commons: Machine learning datasets and tasks for drug discovery and development. *Adv. Neural Inform. Processing Syst. (NeurIPS), Track on Datasets and Benchmarks* (2021).

[39] Weinstein, J. N. et al. The Cancer Genome Atlas Pan-Cancer Analysis Project. *Nat. Genet.* **45**, 1113–1120 (2013). 24071849 10.1038/ng.2764 PMC3919969

[40] Kortuem, K. M. & Stewart, A. K. Carfilzomib. *Blood, J. Am. Soc. Hematol.* **121**, 893–897 (2013). 10.1182/blood-2012-10-459883 23393020

[41] Hummer, A. M., Abanades, B. & Deane, C. M. Advances in computational structure-based antibody design. *Curr. Opin. Struct. Biol.* **74**, 102379 (2022). 35490649 10.1016/j.sbi.2022.102379

[42] Chiu, M., Goulet, D., Teplyakov, A. & Gilliland, G. Antibody structure and function: the basis for engineering therapeutics. *Antibodies (Basel)* **8** 4), (2019). 10.3390/antib8040055 PMC6963682 31816964

[43] Basu, K., Green, E. M., Cheng, Y. & Craik, C. S. Why recombinant antibodies - benefits and applications. *Curr. Opin. Biotechnol.* **60**, 153–158 (2019). 30849700 10.1016/j.copbio.2019.01.012 PMC6728236

[44] Carter, P. J. & Lazar, G. A. Next generation antibody drugs: pursuit of the’high-hanging fruit’. *Nat. Rev. Drug Discov.* **17**, 197–223 (2018). 29192287 10.1038/nrd.2017.227

[45] Beck, A., Goetsch, L., Dumontet, C. & Corvaía, N. Strategies and challenges for the next generation of antibody–drug conjugates. *Nat. Rev. Drug Discov.* **16**, 315–337 (2017). 28303026 10.1038/nrd.2016.268

[46] Saka, K. et al. Antibody design using lstm based deep generative model from phage display library for affinity maturation. *Sci. Rep.* **11**, 5852 (2021). 33712669 10.1038/s41598-021-85274-7 PMC7955064

[47] Kong, X., Huang, W. & Liu, Y. End-to-End Full-Atom Antibody Design. *Proc. Intl. Conf. on Mach. Learn. (ICML),* **202**, 17409–17429 (2023).

[48] Jin, W., Wohlwend, J., Barzilay, R. & Jaakkola, T. Iterative refinement graph neural network for antibody sequence-structure co-design. arXiv preprint arXiv:2110.04624 (2021).

[49] Jin, W., Barzilay, R. & Jaakkola, T. Antibody-Antigen Docking and Design via Hierarchical Structure Refinement. *Proc. 39th Intl. Conf. on Mach. Learn. (ICML),* 162, 10217–10227 (PMLR, 2022).

[50] Luo, S. et al. Antigen-specific antibody design and optimization with diffusion-based generative models for protein structures. *Adv. Neural Inf. Process. Syst.* **35**, 9754–9767 (2022).

[51] Kong, X., Huang, W. & Liu, Y. Conditional antibody design as 3d equivariant graph translation. *arXiv preprint arXiv:2208.06073* (2022).

[52] Zhou, X. et al. Antigen-specific antibody design via direct energy-based preference optimization. *Adv. Neural Inform. Processing Syst. (NeuRIPS),* **37**, 120861–120891 (2024).

[53] Dunbar, J. et al. Sabdab: the structural antibody database. *Nucleic acids Res.* **42**, D1140–D1146 (2014). 24214988 10.1093/nar/gkt1043 PMC3965125

[54] Weber, A., Born, J. & Rodriguez Martínez, M. Titan: T-cell receptor specificity prediction with bimodal attention networks. *Bioinformatics* **37**, i237–i244 (2021). 34252922 10.1093/bioinformatics/btab294 PMC8275323

[55] Jankauskaitė, J., Jiménez-García, B., Dapkūnas, J., Fernández-Recio, J. & Moal, I. H. Skempi 2.0: an updated benchmark of changes in protein–protein binding energy, kinetics and thermodynamics upon mutation. *Bioinformatics* **35**, 462–469 (2019). 30020414 10.1093/bioinformatics/bty635 PMC6361233

[56] Liu, X., Feng, H., Lü, Z. & Xia, K. Persistent tor-algebra for protein–protein interaction analysis. *Brief. Bioinforma.* **24**, bbad046 (2023). 10.1093/bib/bbad046 36790858

[57] Wang, M., Cang, Z. & Wei, G.-W. A topology-based network tree for the prediction of protein–protein binding affinity changes following mutation. *Nat. Mach. Intell.* **2**, 116–123 (2020). 34170981 10.1038/s42256-020-0149-6 PMC7223817

[58] Guo, Z. & Yamaguchi, R. Machine learning methods for protein-protein binding affinity prediction in protein design. *Front. Bioinforma.* **2**, 1065703 (2022). 10.3389/fbinf.2022.1065703 PMC9800603 36591334

[59] Berman, H. M. et al. The Protein Data Bank. *Nucleic Acids Res.* **28**, 235–242 (2000). 10592235 10.1093/nar/28.1.235 PMC102472

[60] Xu, M. et al. Peer: a comprehensive and multi-task benchmark for protein sequence understanding. *Adv. Neural Inf. Process. Syst.* **35**, 35156–35173 (2022).

[61] Gilson, M. K. et al. BindingDB in 2015: A public database for medicinal chemistry, computational chemistry and systems pharmacology. *Nucleic Acids Res* **44**, D1045–53 (2015). 26481362 10.1093/nar/gkv1072 PMC4702793

[62] Mason, D. M. et al. Optimization of therapeutic antibodies by predicting antigen specificity from antibody sequence via deep learning. *Nat. Biomed. Eng.* **5**, 600–612 (2021). 33859386 10.1038/s41551-021-00699-9

[63] Jing, H. et al. Accurate prediction of antibody function and structure using bio-inspired antibody language model. *Brief. Bioinforma.* **25**, bbae245 (2024). 10.1093/bib/bbae245 PMC11128484 38797969

[64] Bennett, N. R. et al. Atomically accurate de novo design of antibodies with rfdiffusion. *bioRxiv* https://www.biorxiv.org/content/early/2025/02/28/2024.03.14.585103.full.pdf (2025). 10.1038/s41586-025-09721-5 PMC12727541 41193805

[65] Yin, R. & Pierce, B. G. Evaluation of alphafold antibody-antigen modeling with implications for improving predictive accuracy. *Protein Sci.* **33**, e4865 (2024). 38073135 10.1002/pro.4865 PMC10751731

[66] Schneider, C., Raybould, M. I. J. & Deane, C. M. Sabdab in the age of biotherapeutics: updates including sabdab-nano, the nanobody structure tracker. *Nucleic Acids Res.* **50**, D1368–D1372 (2021). 10.1093/nar/gkab1050 PMC8728266 34986602

[67] Consortium, T. U. UniProt: the Universal Protein Knowledgebase in 2023. *Nucleic Acids Res.* **51**, D523–D531 (2022). 10.1093/nar/gkac1052 PMC9825514 36408920

[68] Madani, A. et al. Progen: Language modeling for protein generation. *arXiv preprint arXiv:2004.03497* (2020).

[69] Bunne, C. et al. How to build the virtual cell with artificial intelligence: Priorities and opportunities. *Cell* **187**, 7045–7063 (2024). 39672099 10.1016/j.cell.2024.11.015 PMC12148494

[70] Song, L., Segal, E. & Xing, E. Toward ai-driven digital organism: Multiscale foundation models for predicting, simulating and programming biology at all levels. *arXiv preprint arXiv:2412.06993* (2024).

[71] Barkan, E. et al. Leveraging large language models to predict antibody biological activity against influenza A hemagglutinin. *Computational Struct. Biotechnol. J.* **27**, 1286–1295 (2025). 10.1016/j.csbj.2025.03.038 PMC11995015 40230408

[72] Gopalan, S. & Narayanan, S. Hallucinations in alphafold3 for intrinsically disordered proteins with disorder in biological process residues. *arXiv preprint arXiv:2510.15939* (2025).

[73] Thomas, S. D. et al. Impact of intrinsically disordered regions and functional disorder hotspots in the human kinome. *Brief. Bioinforma.* **26**, bbaf662 (2025). 10.1093/bib/bbaf662 PMC12696717 41378882

[74] Pinet, L. et al. Structural and dynamic characterization of the c-terminal tail of ErbB2: Disordered but not random. *Biophysical J.* **120**, 1869–1882 (2021). 10.1016/j.bpj.2021.03.005 PMC8204338 33741354

[75] Mehdiabadi, M. et al. Critical assessment of protein intrinsic disorder round 3-predicting disorder in the era of protein language models. *Proteins: Struct., Funct., Bioinforma.* **94**, 414–424 (2026). 10.1002/prot.70045 PMC12750029 40859602

[76] Bolton, E. et al. Biomedlm: A 2.7 b parameter language model trained on biomedical text. *arXiv preprint arXiv:2403.18421* (2024).

[77] Zhang, O. et al. Graph neural networks in modern ai-aided drug discovery. *Chem. Rev.* **125**, 10001–10103 (2025). 40959983 10.1021/acs.chemrev.5c00461

[78] Rives, A. et al. Biological structure and function emerge from scaling unsupervised learning to 250 million protein sequences. *Proc. Natl. Acad. Sci.* **118**, e2016239118 (2021). 33876751 10.1073/pnas.2016239118 PMC8053943

[79] Zhou, G. et al. Uni-mol: A universal 3d molecular representation learning framework. In *The eleventh international conference on learning representations* (2023).

[80] Wang, J. et al. Token-mol 1.0: tokenized drug design with large language models. *Nat. Commun.* **16**, 4416 (2025). 40360500 10.1038/s41467-025-59628-y PMC12075800

[81] Hayes, T. et al. Simulating 500 million years of evolution with a language model. *bioRxiv* https://www.biorxiv.org/content/early/2024/07/02/2024.07.01.600583.full.pdf (2024). 10.1126/science.ads0018 39818825

[82] Zhao, H. et al. Protein–peptide docking with a rational and accurate diffusion generative model. *Nat. Mach. Intell.* **7**, 1308–1321 (2025).

[83] Watson, J. L. et al. De novo design of protein structure and function with rfdiffusion. *Nature* **620**, 1089–1100 (2023). 37433327 10.1038/s41586-023-06415-8 PMC10468394

[84] Hoogeboom, E., Satorras, V. G., Vignac, C. & Welling, M. Equivariant diffusion for molecule generation in 3d (2022). arxiv: 2203.17003

[85] Gridach, M., Nanavati, J., Abidine, K. Z. E., Mendes, L. & Mack, C. Agentic AI for scientific discovery: A survey of progress, challenges, and future directions. *arXiv preprint arXiv:2503.08979* (2025).

[86] Ramos, M. C., Collison, C. J. & White, A. D. A review of large language models and autonomous agents in chemistry. *Chem. Sci.* **16**, 2514–2572 (2025). 10.1039/d4sc03921a PMC11739813 39829984

[87] Vaswani, A. et al. Attention is all you need. *Adv. Neural Inform. Processing Syst.* (NeurIPS), **30**, 5998–6008 (2017).

[88] Raffel, C. et al. Exploring the limits of transfer learning with a unified text-to-text transformer. *J. Mach. Learn. Res.* **21**, 1–67 (2020).34305477

[89] Chen, B. et al. xtrimopglm: Unified 100b-scale pre-trained transformer for deciphering the language of protein (2024). arXiv: 2401.06199

[90] Pei, Q. et al. Biot5: Enriching cross-modal integration in biology with chemical knowledge and natural language associations. In *Proc. 2023 Conference on Empirical Methods in Natural Language Processing (EMNLP),* 1102–1123 (2023).

[91] Pei, Q. et al. Biot5+: Towards generalized biological understanding with iupac integration and multi-task tuning. *Findings of ACL 2024,* 1216–1240 (2024).

[92] Chaves, J. M. Z. et al. Tx-llm: A large language model for therapeutics. *arXiv preprint arXiv:2406.06316* (2024).

[93] Touvron, H. et al. Llama: Open and efficient foundation language models. arXiv: 2302.13971 (2023).

[94] Born, J. & Manica, M. Regression transformer enables concurrent sequence regression and generation for molecular language modelling. *Nat. Mach. Intell.* **5**, 432–444 (2023).

[95] Zausinger, J. et al. Regress, don’t guess – a regression-like loss on number tokens for language models (2024). arXiv: 2411.02083

[96] van den Oord, A., Vinyals, O. & Kavukcuoglu, K. Neural discrete representation learning (2018). arXiv: 1711.00937

[97] Xiong, P., Zhang, C., Zheng, W. & Zhang, Y. Bindprofx: assessing mutation-induced binding affinity change by protein interface profiles with pseudo-counts. *J. Mol. Biol.* **429**, 426–434 (2017). 27899282 10.1016/j.jmb.2016.11.022 PMC5963940

[98] Olsen, T. H., Boyles, F. & Deane, C. M. Observed antibody space: A diverse database of cleaned, annotated, and translated unpaired and paired antibody sequences. *Protein Sci.* **31**, 141–146 (2022). 34655133 10.1002/pro.4205 PMC8740823

[99] Suzek, B. E. et al. UniRef clusters: a comprehensive and scalable alternative for improving sequence similarity searches. *Bioinformatics* **31**, 926–932 (2015). 25398609 10.1093/bioinformatics/btu739 PMC4375400

[100] Tingle, B. I. et al. Zinc-22-a free multi-billion-scale database of tangible compounds for ligand discovery. *J. Chem. Inf. Modeling* **63**, 1166–1176 (2023). 10.1021/acs.jcim.2c01253 PMC9976280 36790087

[101] Kim, S. et al. PubChem 2023 update. *Nucleic Acids Res.* **51**, D1373–D1380 (2022). 10.1093/nar/gkac956 PMC9825602 36305812

[102] Szklarczyk, D. et al. The STRING database in 2023: protein-protein association networks and functional enrichment analyses for any sequenced genome of interest. *Nucleic Acids Res.* **51**, D638–D646 (2022). 10.1093/nar/gkac1000 PMC9825434 36370105

[103] Biology, C. S.-C. et al. Cz cellxgene discover: A single-cell data platform for scalable exploration, analysis and modeling of aggregated data. *BioRxiv* 2023–10 10.1093/nar/gkae1142 (2023). 10.1093/nar/gkae1142 PMC11701654 39607691

[104] Jin, R. et al. Attabseq: an attention-based deep learning prediction method for antigen–antibody binding affinity changes based on protein sequences. *Brief. Bioinforma.* **25**, bbae304 (2024). 10.1093/bib/bbae304 PMC11221889 38960407

