# Supplementary material for: MAMMAL - Molecular Aligned Multi-Modal Architecture and Language for biomedical discovery
Source: NPJ Drug Discov. 2026 May 4;3:14. doi: 10.1038/s44386-026-00047-4 (PMC13267068; doi:10.1038/s44386-026-00047-4)
Supplement: Supplementary file 1 — Supplementary information [file 44386_2026_47_MOESM1_ESM.pdf]

MAMMAL - Molecular Aligned Multi-Modal Architecture and Language for Biomedical Discovery

Yoel Shoshan[1*†], Moshiko Raboh[1†], Michal Ozery-Flato[1†], Vadim Ratner[1], Alex Golts[1], Jeffrey K. Weber[2], Ella Barkan[1], Simona Rabinovici-Cohen[1], Sagi Polaczek[1], Ido Amos[1], Ben Shapira[1], Liam Hazan[1], Matan Ninio[1], Sivan Ravid[1], Michael M. Danziger[1], Yosi Shamay[3], Sharon Kurant[3], Joseph A. Morrone[2], Parthasarathy Suryanarayanan[2], Michal Rosen-Zvi[1†], Efrat Hexter[1†]

[1]IBM Research-Israel, IBM Research, Haifa, 3498825, Israel.
[2]IBM TJ Watson Research Center, IBM Research, 1101 Kitchawan Rd., NY, 10598, Yorktown Heights, USA.
[3]Faculty of Biomedical Engineering, Technion - IIT, 3200003, Haifa, Israel.

*Corresponding author(s). E-mail(s): yoels@il.ibm.com;
†These authors contributed equally to this work.

Supplementary Information

1 Architecture - Additional Details

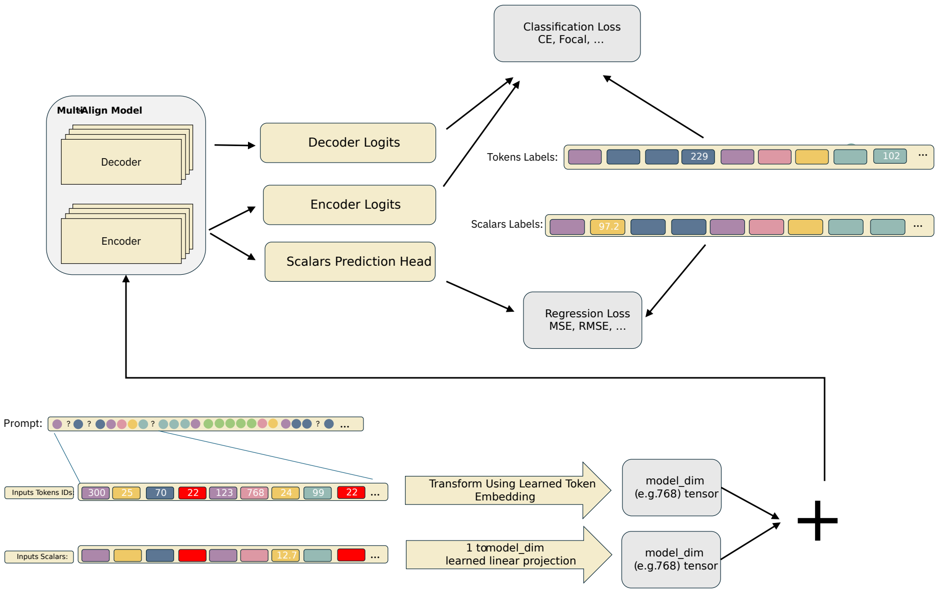

Fig. S1 A prompt, consisting of both token IDs and scalars is processed and enters the encoder. Both the encoder and the decoder output logits which are used for classification loss. Additionally, the output of the encoder is sent to a (learned) scalars prediction head which allows the prediction scalars for any subset of the tokens, and is used in the regression loss. In this illustration, a single scalar input ("12.7") is being used, and a single scalar's outputs are predicted by the model ("97.2"). However, the method fully supports an arbitrary number of input scalars and outputs. Visualization created using PowerPoint.

One of the key aspects in the MAMMAL method is the built-in support for scalar inputs and outputs. Figure S1 illustrates how this is achieved.

A user prompt, usually expressed as a single text line, is processed into two input sequences: a. Input token IDs, which are a sequence of integer values representing tokens in the vocabulary, and b. a sequence of inputs scalars (by convention, containing NaNs for positions for which no input scalar is provided).

The input tokens IDs are transformed using a learned token embedding, and the input scalars are transformed using a learned linear transformation that projects each single scalar element into the model dimension (e.g., 768).

Both representations are added (not concatenated) and fed into the encoder stack. Using this approach, both tasks that use encoder-only mode and encoder-decoder mode benefit from the ability to get as input an arbitrary number of scalars (at most as many as the number of tokens that are being fed in).

For scalars outputs (gene expression, binding free energy, etc.), the encoder stack has an additional prediction head, which outputs a scalar value for every input element. How to deal with scalar outputs in locations where there is no scalar label is the user's choice, but the default is to ignore those.

The support of scalar outputs in the encoder-decoder mode is an improvement that we intend to add in future generations of the model/method.

2 Prompt Syntax

A typical prompt is built as a combination of entities of the following types:

- **Sequence** : A sequence of amino acids or other chemical representations, such as SMILES, which may encompass a full sequence or a specific region.
- Molecule: A complete molecule, such as a protein chain or small molecule, which may contain multiple sequences corresponding to sub-regions within the molecule. Each molecule is initiated with two special tokens - a general token indicating the entity's hierarchical level, ⟨MOLECULAR_ENTITY⟩, followed by a token specifying the molecule type (e.g., ⟨MOLECULAR_ENTITY_EPITOPE⟩). Additionally, molecules can be marked with natural start and end tokens to denote instances where truncation has occurred, either in the original database or due to sequence length constraints.
- MolecularSystem: A quaternary structure consisting of multiple molecules, denoted by the ⟨COMPLEX_ENTITY⟩ special token.
- GlobalSystem: A system comprising multiple interacting MolecularSystem entities.
- Attribute: A representation of properties or interactions among the entities.

To support a wide range of tasks, MAMMAL utilizes special placeholder tokens of the form ⟨SENTINEL_ID_?⟩. These tokens serve as positionally aware anchors in the decoder output and are designed to flexibly handle both structured and unstructured prediction targets. For complex generation tasks, such as sequence infilling, molecule editing, or antibody design, multiple sentinel tokens (e.g., ⟨SENTINEL_ID_0⟩, ⟨SENTINEL_ID_1⟩) allow the model to generate multiple, disjoint segments of output. This enables fine-grained control over where the model should insert or complete information.

In simpler settings such as classification or regression, a single sentinel token (e.g., ⟨SENTINEL_ID_0⟩) is used to indicate the position where the model should output the prediction. This unified approach allows all tasks, whether discrete or continuous, generative or discriminative, to be framed consistently as a form of sequence-to-sequence learning, while preserving flexibility across domains. The use of sentinel tokens thus enables seamless task specification and modular decoding, making the model architecture adaptable to a broad spectrum of biomedical problems.

Example 1: Illustrated in Figure S2. Given two interacting molecules – variable region of a TCR beta chain of an unspecified organism and an epitope of organism 567, find whether they bind, and to which organism the first molecule belongs.:

- encoder inputs = ⟨@TOKENIZER-TYPE=AA⟩ ⟨BINDING_AFFINITY_CLASS⟩ ⟨SENTINEL_ID_0⟩ ⟨MOLECULAR_ENTITY⟩⟨MOLECULAR_ENTITY_TCR_BETA_VDJ⟩ ⟨ATTRIBUTE_ORGANISM⟩⟨SENTINEL_ID_1⟩ AC...DF ⟨MOLECULAR_ENTITY⟩ ⟨MOLECULAR_ENTITY_EPITOPE⟩⟨ATTRIBUTE_ORGANISM⟩⟨5⟩⟨6⟩⟨7⟩⟨8⟩ LM...VW ⟨EOS⟩
- labels = ⟨@TOKENIZER-TYPE=AA⟩⟨SENTINEL_ID_0⟩⟨1⟩⟨SENTINEL_ID_1⟩⟨2⟩⟨3⟩⟨4⟩ ⟨EOS⟩

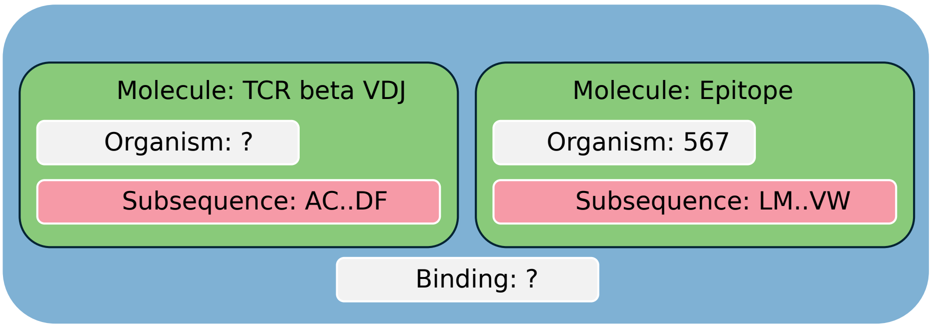

Fig. S2 Entity hierarchy for the task of binding prediction of two proteins, and organism prediction of the first one. Visualization created using PowerPoint.

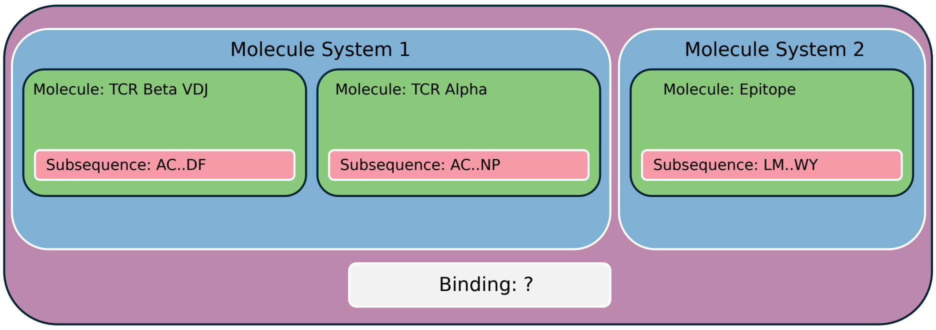

Fig. S3 Entity hierarchy for the task of binding prediction of a TCR and an epitope. "Molecule System 1" represents the TCR complex, "Molecule System 2" represents the antigen, and the entire prompt represents their interaction. Visualization created using PowerPoint.

Example 2 : Illustrated in Figure S3. Given a complex entity, T-cell receptor consisting of alpha and beta chains, and an epitope, predict if they bind.:

- encoder inputs = ⟨@TOKENIZER-TYPE=AA⟩⟨BINDING_AFFINITY_CLASS⟩ ⟨SENTINEL_ID_0⟩ ⟨COMPLEX_ENTITY⟩⟨MOLECULAR_ENTITY⟩⟨MOLECULAR_ENTITY_TCR_BETA_VDJ⟩ AB...DF ⟨MOLECULAR_ENTITY⟩⟨MOLECULAR_ENTITY_TCR_ALPHA⟩ AC...NP ⟨COMPLEX_ENTITY⟩ ⟨MOLECULAR_ENTITY⟩⟨MOLECULAR_ENTITY_EPITOPE⟩LM...WY ⟨EOS⟩
- labels = ⟨@TOKENIZER-TYPE=AA⟩⟨SENTINEL_ID_0⟩⟨1⟩⟨EOS⟩

Example: Given two binding chains – TCR beta chain and an epitope, unmask 3 spans within the beta chain:

- labels = ⟨@TOKENIZER-TYPE=AA⟩⟨SENTINEL_ID_0⟩AC⟨SENTINEL_ID_1⟩ AD⟨SENTINEL_ID_2⟩CD⟨EOS⟩

- encoder inputs = ⟨`@TOKENIZER-TYPE=AA`⟩ ⟨`BINDING_AFFINITY_CLASS`⟩⟨`1`⟩ ⟨`MOLECULAR_ENTITY`⟩⟨`MOLECULAR_ENTITY_EPITOPE`⟩⟨`SEQUENCE_NATURAL_START`⟩ LM. . . WY⟨`SEQUENCE_NATURAL_END`⟩⟨`MOLECULAR_ENTITY`⟩ ⟨`MOLECULAR_ENTITY_TCR_BETA_VDJ`⟩ ⟨`SENTINEL_ID_0`⟩**C. . . D**⟨`SENTINEL_ID_1`⟩ **DF**⟨`SENTINEL_ID_2`⟩**FDF** ⟨`EOS`⟩

Example 3: (for multitokenizer): DTI

- labels = ⟨`@TOKENIZER-TYPE=AA`⟩⟨`SENTINEL_ID_0`⟩⟨`1`⟩⟨`EOS`⟩
- encoder inputs = ⟨`@TOKENIZER-TYPE=AA`⟩⟨`BINDING_AFFINITY_CLASS`⟩ ⟨`SENTINEL_ID_0`⟩⟨`@TOKENIZER-TYPE=SMILES@MAX-LEN=10`⟩⟨`MOLECULAR_ENTITY`⟩ ⟨`MOLECULAR_ENTITY_SMALL_MOLECULE`⟩**CCC=CCC** ⟨`@TOKENIZER-TYPE=AA@MAXLEN=15`⟩⟨`MOLECULAR_ENTITY`⟩ ⟨`MOLECULAR_ENTITY_EPITOPE`⟩ **LMNPQRSTUVWY** ⟨`EOS`⟩

Examples showing how a prompt is created for several downstream tasks can be found in Table S1.

2.1 Modular Tokenizer and Meta Tokens

To support multiple modalities within a single prompt, we developed "Modular Tokenizer" which allows to utilization of different tokenizers within a single prompt by mapping tokens from different domains (like SMILES carbon "C" and amino acid cysteine "C") to the same ID space.

We use meta tokens of the format ⟨`@TOKENIZER-TYPE=...`⟩ to indicate that everything following this meta token, up to the next meta token (or the end of the prompt) should be tokenized with the defined tokenizer. For example, ⟨`@TOKENIZER-TYPE=AA`⟩ tokenizes amino-acids, while ⟨`@TOKENIZER-TYPE=SMILES`⟩ can tokenize SMILES. Since all of those "sub tokenizers" must exist in a single vocabulary space, our modular tokenizer orchestrates that, and provides mechanism to avoid conflicts and allow for sub-tokenizers to co-exist. Additionally, we support additional instructions within a meta token, beyond just the expression of which (sub) tokenizer should be used. For example, ⟨`@TOKENIZER-TYPE=AA@MAX-LEN=1000`⟩ allows users to restrict the maximum length of the tokenized sequence, which provides more granular control compared to only controlling the overall total max sequence tokenized length. It is worth emphasizing that meta tokens, by themselves, do not get tokenized into any token. They serve as instructions for the modular tokenizer. Further details on the implementation can be found on https://github.com/BiomedSciAI/fuse-med-ml/tree/master/fuse/data/tokenizers/modular_tokenizer

3 Pretraining Details

3.1 Infrastructure

`ibm/biomed.omics.bl.sm.ma-ted-458m` model was trained on an OpenShift cluster. It was trained for three months over two nodes with 16 A100-80G GPUs. The training framework was implemented using FuseMedML [1] and PyTorch, with distributed processing supported by PyTorch Fully Sharded Data Parallel (FSDP) for efficient parallelism.

3.2 Hyperparameters

We train `ibm/biomed.omics.bl.sm.ma-ted-458m` using AdamW optimizer, with the following hyperparameters: $\beta1 = 0.9$, $\beta2 = 0.999$. We use a weight decay of 0.01 and a gradient clipping norm of 1.0. We employ 2K warmup steps until reaching the maximum learning rate and utilize a cosine decay scheduler to decay LR to 10% of the maximum learning rate by the end of training. The maximum sequence length was set per task to be effective yet efficient. When required, instead of naively truncating the end of a sequence, we first wrapped the sequence with special start and end tokens to provide a hint for the model as to whether the beginning or end of a sequence was truncated. Then we randomly cut a random sub-sequence with the required length. Batch sizes were tuned per task given the maximum sequence length to maximize GPU memory utilization.

3.3 Datasets

`ibm/biomed.omics.bl.sm.ma-ted-458m` was pre-trained using six diverse datasets spanning multiple domains.

UniRef90 [2], one of the clustered databases in UniProt [3] (UniProt Reference Clusters), groups protein sequences that share at least 90% identity and 80% sequence overlap with the longest sequence in each cluster (the seed sequence). This clustering approach reduces redundancy while preserving the diversity of functional sequences, providing a rich protein dataset.

OAS [4] (Observed Antibody Space) offers unpaired antibody sequences, specifically focusing on the variable regions of light or heavy chains. After filtering for sequences with complete variable domains and retaining only the sequences with standard amino acids, we finalized a dataset of approximately 650 million sequences. Each sequence is annotated with its chain type (heavy or light) and species information.

STRING [5] is a database that integrates data from experimental findings, computational predictions, and text mining to describe protein-protein interactions. We curated a dataset of 390 million positive protein interaction pairs, considering only pairs that had a STRING confidence score above 500. Additionally, we curated 390 million pseudo-negative pairs by randomly matching proteins from the same species, resulting in a second dataset of 780 million samples.

CELLxGENE [6], a platform for single-cell transcriptomics data, was used to assemble a dataset of gene expression sequences from individual cells. After filtering for human samples labeled as "cell" in the 'suspension_type' field, we curated a dataset

of 30 million samples. The sequences for model training were created as ranked lists of gene names, similar to Geneformer [7], but with several differences. Instead of normalizing by median expression and ranking the now unique values of expression count, we log-normalized and binned the samples, followed by alphabetic sorting to make the task well-defined. The binning removes the ability of noisy reads to significantly affect the pretraining and alphabetic sorting ensures that for every masked token there is a unique correct answer.

For small-molecule data, we utilized two main sources: (1) **PubChem** [8], a comprehensive chemical database maintained by NCBI, and (2) **ZINC22** [9], a large library of drug-like molecules. From PubChem, we curated a subset of 80 million "drug-like" molecules, removing duplicates and following Lipinski's rule of five to ensure drug-likeness. Additionally, we sampled 120 million molecules from the ZINC22 database, focusing on small molecules with fewer than 30 heavy atoms to ensure dense coverage of "drug-like" chemical space. This led to a final dataset of 200 million small-molecule examples.

3.4 Multitask Pretraining Approach

`ibm/biomed.omics.bl.sm.ma-ted-458m` was pre-trained on seven tasks simultaneously using a multitask learning approach. Gradients from each task were aggregated before updating the optimizer. This approach, combined with a custom query system, enables the model to learn from different tasks and domains during co-training.

3.5 Pretraining Tasks

Language Model Tasks. Four language model tasks were defined: (1) amino-acid sequence representation of antibodies based on OAS database [4]; (2) amino-acid sequence representation of general proteins based on Uniref90 [2, 3]; (3) SMILES representation of small molecules based on a mixture of PubChem [8] and Zinc databases [9]; and (4) Geneformer format representations [7] of cell genes based on CELLxGENE [6]. In all language modeling tasks, we employed span-denoising (similar to T5 [10]) with a mean noise span length of 5 and a noise density of 0.15. Additionally, a special token was introduced per entity type to make the model aware of it (e.g., ⟨`MOLECULAR_ENTITY_TYPE_ANTIBODY_HEAVY_CHAIN`⟩). When sequences were available from different species, an additional special token was also introduced (e.g., ⟨`ATTRIBUTE_ORGANISM_HUMAN`⟩)

Antibody Denoise. This task focuses on recovering corrupted antibodies, represented by amino acid sequences. The corrupted sequence is generated by first sampling a value t from the range [1, 500], and then uniformly corrupting the amino acid tokens with a probability proportional to t. The antibody sequences used in this task are sourced from the OAS (Observatory of Antibody Space) dataset.

Protein-Protein Interaction. As part of the pretraining process, two tasks were defined for learning protein-protein interactions: a classification task and a generation task, both utilizing data from the STRING database [5]. Interactions with a score higher than 500 are labeled as positive, while random pairs of proteins are treated as negative interactions. For the classification task, a balanced dataset comprising both

positive and negative pairs is used. In the generation task, the model is trained on positive pairs only, where it learns to generate an interacting protein given an input protein.

4 Additional Results

Table S1: Examples of Encoder Inputs and Decoder Outputs for Benchmarks

Bench.	Encoder input	Decoder label
Cell type	⟨@TOKENIZER-TYPE=GENE⟩⟨MOLECULAR_ENTITY⟩⟨MOLECULAR_ENTITY_CELL_GENE_EXPRESSION_RANKED⟩ [MALAT1] [RPL10] ... [ZNF136] [ZNF514] ⟨CELL_TYPE_CLASS⟩⟨SENTINEL_ID_0⟩⟨EOS⟩	⟨@TOKENIZER-TYPE=CELL_ATTRIBUTES⟩⟨SENTINEL_ID_0⟩**[CL:0001062]** ⟨EOS⟩
BBBP	⟨@TOKENIZER-TYPE=SMILES⟩⟨MOLECULAR_ENTITY⟩⟨MOLECULAR_ENTITY_SMALL_MOLECULE⟩⟨BBBP⟩⟨SENTINEL_ID_0⟩ ⟨@TOKENIZER-TYPE=SMILES@MAX-LEN=2100⟩ C(Cl)Cl ⟨EOS⟩	⟨@TOKENIZER-TYPE=SMILES⟩ ⟨SENTINEL_ID_0⟩⟨1⟩⟨EOS⟩
ClinTox Toxic	⟨@TOKENIZER-TYPE=SMILES⟩⟨MOLECULAR_ENTITY⟩⟨MOLECULAR_ENTITY_SMALL_MOLECULE⟩⟨TOXICITY⟩⟨SENTINEL_ID_0⟩ ⟨@TOKENIZER-TYPE=SMILES@MAX-LEN=2100⟩ C#CC1(CCCCC1)OC(=O)N ⟨EOS⟩	⟨@TOKENIZER-TYPE=SMILES⟩ ⟨SENTINEL_ID_0⟩⟨0⟩⟨EOS⟩
ClinTox FDA Approval	⟨@TOKENIZER-TYPE=SMILES⟩⟨MOLECULAR_ENTITY⟩⟨MOLECULAR_ENTITY_SMALL_MOLECULE⟩⟨FDA_APPR⟩⟨SENTINEL_ID_0⟩ ⟨@TOKENIZER-TYPE=SMILES@MAX-LEN=2100⟩ C#CC1(CCCCC1)OC(=O)N ⟨EOS⟩	⟨@TOKENIZER-TYPE=SMILES⟩ ⟨SENTINEL_ID_0⟩⟨1⟩⟨EOS⟩
Cancer-Drug Response	⟨@TOKENIZER-TYPE=SCALARS_LITERALS⟩ <MASK > ⟨@TOKENIZER-TYPE=SMILES⟩ ⟨MOLECULAR_ENTITY⟩⟨MOLECULAR_ENTITY_SMALL_MOLECULE⟩⟨SMILES_SEQUENCE⟩ CN(C)CCOc... [nH]2)cc1 ⟨@TOKENIZER-TYPE=GENES⟩⟨MOLECULAR_ENTITY⟩⟨MOLECULAR_ENTITY_CELL_GENE_EXPRESSION_RANKED⟩ [B2M] [RPL10] ... [ZBTB16] [ZNF429] ⟨EOS⟩	⟨@TOKENIZER-TYPE=SCALARS_LITERALS⟩3.966226 ⟨@TOKENIZER-TYPE=SMILES⟩ ... [ZBTB16] [ZNF429] ⟨EOS⟩

Continued on next page

Table S1 – continued from previous page

Bench.	Encoder input	Decoder label
Antibody design	⟨@TOKENIZER-TYPE=AA⟩⟨COMPLEX_ENTITY⟩ ⟨ATTRIBUTE_ORGANISM⟩⟨ATTRIBUTE_ORGANISM_HUMAN⟩⟨MOLECULAR_ENTITY⟩⟨MOLECULAR_ENTITY_TYPE_ANTIBODY_LIGHT_CHAIN⟩ AB...CD⟨SENTINEL_ID_0⟩GF...KL⟨SENTINEL_ID_1⟩...TC ⟨MOLECULAR_ENTITY⟩⟨MOLECULAR_ENTITY_TYPE_ANTIBODY_HEAVY_CHAIN⟩ AA...DD⟨SENTINEL_ID_2⟩FK...KF⟨SENTINEL_ID_3⟩...JJ ⟨MOLECULAR_ENTITY⟩⟨MOLECULAR_ENTITY_EPITOPE⟩AB...GK ⟨EOS⟩	⟨@TOKENIZER-TYPE=AA⟩ ⟨SENTINEL_ID_0⟩ ABC⟨SENTINEL_ID_1⟩ DDDDD⟨SENTINEL_ID_2⟩ EEFF...⟨SENTINEL_ID_3⟩ GKGK⟨EOS⟩
AbAg Bind	⟨@TOKENIZER-TYPE=AA⟩ ⟨BINDING_AFFINITY_CLASS⟩⟨SENTINEL_ID_0⟩ ⟨@TOKENIZER-TYPE=AA@MAX-LEN=700⟩ ⟨MOLECULAR_ENTITY⟩⟨MOLECULAR_ENTITY_ANTIBODY_HEAVY_CHAIN⟩ EVQ...KSC ⟨@TOKENIZER-TYPE=AA@MAX-LEN=700⟩ ⟨MOLECULAR_ENTITY⟩⟨MOLECULAR_ENTITY_ANTIGEN⟩ MEL...YEG ⟨EOS⟩	⟨@TOKENIZER-TYPE=AA⟩ ⟨SENTINEL_ID_0⟩⟨1⟩⟨EOS⟩
TCR Bind	⟨@TOKENIZER-TYPE=AA⟩ ⟨BINDING_AFFINITY_CLASS⟩⟨SENTINEL_ID_0⟩ ⟨@TOKENIZER-TYPE=AA@MAX-LEN=700⟩ ⟨MOLECULAR_ENTITY⟩⟨MOLECULAR_ENTITY_TCR_BETA_VDJ⟩ TIQ...TVV ⟨@TOKENIZER-TYPE=AA@MAX-LEN=170⟩ ⟨MOLECULAR_ENTITY⟩⟨MOLECULAR_ENTITY_EPITOPE⟩ LEPLVDLPI ⟨EOS⟩	⟨@TOKENIZER-TYPE=AA⟩ ⟨SENTINEL_ID_0⟩⟨1⟩⟨EOS⟩
PPI $\Delta\Delta G$	⟨@TOKENIZER-TYPE=AA⟩⟨GENERAL_AFFINITY_CLASS⟩⟨@TOKENIZER-TYPE=SCALARS_LITERALS⟩<MASK> ⟨@TOKENIZER-TYPE=AA⟩⟨COMPLEX_ENTITY⟩ ⟨MOLECULAR_ENTITY⟩⟨MOLECULAR_ENTITY_GENERAL_PROTEIN⟩IS...VY ⟨@TOKENIZER-TYPE=AA⟩⟨COMPLEX_ENTITY⟩ ⟨MOLECULAR_ENTITY⟩⟨MOLECULAR_ENTITY_GENERAL_PROTEIN⟩DC...KCNF ...KC ⟨@TOKENIZER-TYPE=AA⟩⟨MUTATED⟩ ⟨MOLECULAR_ENTITY⟩⟨MOLECULAR_ENTITY_GENERAL_PROTEIN⟩DC...KCQF ...KC ⟨EOS⟩	⟨@TOKENIZER-TYPE=AA⟩ ⟨GENERAL_AFFINITY_CLASS⟩ ⟨@TOKENIZER-TYPE=SCALARS_LITERALS⟩ 0.244⟨@TOKENIZER-TYPE=AA⟩ ⟨COMPLEX_ENTITY⟩ ...KC ⟨EOS⟩

Continued on next page

Table S1 – continued from previous page

Bench.	Encoder input	Decoder label
DTI	⟨@TOKENIZER-TYPE=AA⟩<MASK> ⟨@TOKENIZER-TYPE=AA⟩⟨MOLECULAR_ENTITY⟩ ⟨MOLECULAR_ENTITY_GENERAL_PROTEIN⟩ **AD...**⟨@TOKENIZER-TYPE=SMILES⟩ ⟨MOLECULAR_ENTITY⟩ ⟨MOLECULAR_ENTITY _SMALL_MOLECULE⟩ **CC=...** ⟨EOS⟩	⟨@TOKENIZER-TYPE=SCALARS _LITERALS⟩ {standardized pKd}

Table S1: Examples (per fine-tune task) of encoder input ("prompt") and Decoder label (ground truth labels). "..." is used when part of the sequence text is not shown, for brevity and readability. For example "QG...SF" instead of "QGCQVVQGN-LELTYLPTNASLSF"

Table S2 Cell type additional results

model	Accuracy	F1	Precision	Recall
scBERT	0.759	0.691	N/A	N/A
CIForm	0.820	0.710	N/A	N/A
MAMMAL	**0.856±0.004**	**0.763±0.012**	0.774±0.016	0.761±0.011

Table S3 Statistics of GDSC Datasets

Dataset	# Cell lines	# Drugs	# Cell-Drug pairs
Cancer-Drug Resp.1	958	208	177K
Cancer-Drug Resp.2	805	137	92K
Cancer-Drug Resp.3	561	223	107K

Table S4 Ab Infilling additional results

model	CDRH1-AAR	CDRH2-AAR	CDRH3-AAR	CDRL1-AAR	CDRL2-AAR	CDRL3-AAR
dyMEAN [11]	0.757	0.685	0.375	0.755	0.831	0.521
MAMMAL	**0.832 ±0.003**	**0.742 ±0.012**	**0.446 ±0.002**	**0.780 ±0.017**	**0.844 ±0.012**	**0.724 ±0.010**

5 Additional results - AlphaFold3

Table S5 Performance comparison between MAMMAL (pre-trained and fine-tuned) and various AlphaFold3 (AF3) configurations for antibody-antigen binding prediction. The metrics are Area Under the Receiver Operating Characteristic curve (AUROC) and Area Under the Precision-Recall Curve (AUPRC)

Method	AUROC	AUPRC
MAMMAL Pre Trained	0.530	0.509
MAMMAL Fine Tuned	**0.881**	**0.915**
AF3 Heavy chain only ipTM	0.448	0.489
AF3 Heavy chain only pTM	0.496	0.514
AF3 Heavy+Light chains ipTM	0.504	0.519
AF3 Heavy+Light chains pTM	0.497	0.519
Control: AF3 H CD206 ipTM	0.455	0.5
Control: AF3 H CD206 pTM	0.66	0.583

Table S6 AUROC performance comparison of nanobody–antigen binding prediction on six targets.

Method	Albumin	CD206	EGFR	TBG	TNFα	VWF
MAMMAL Fine Tuned	0.914	0.997	0.941	0.63	0.857	0.832
MAMMAL Pre Trained	0.428	0.478	0.406	0.644	0.663	0.506
AF3 ipTM	0.586	0.587	0.487	1.0	0.874	0.324
AF3 pTM	0.578	0.39	0.428	0.926	0.824	0.46

Table S7 AUPRC performance comparison of nanobody–antigen binding prediction on six targets.

Method	Albumin	CD206	EGFR	TBG	TNFα	VWF
MAMMAL Fine Tuned	0.882	0.995	0.955	0.219	0.751	0.802
MAMMAL Pre Trained	0.417	0.359	0.395	0.237	0.553	0.464
AF3 ipTM	0.587	0.483	0.436	1.0	0.81	0.305
AF3 pTM	0.551	0.331	0.377	0.859	0.739	0.367

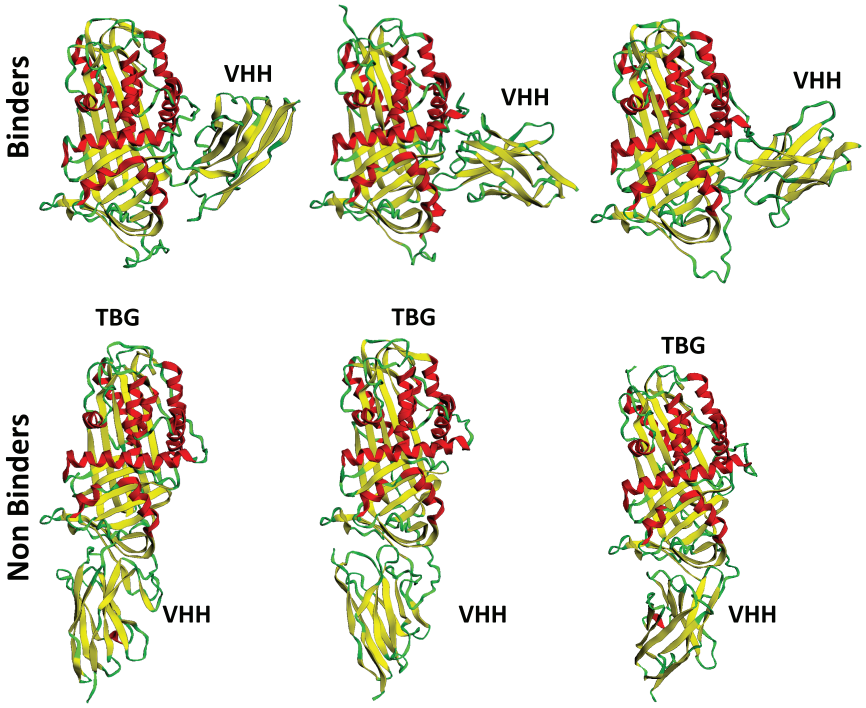

Fig. S4 TBG and three binding and three non-binding VHHs (nanobodies) as predicted by AF3. Visualizations generated using PyMOL.

6 MAMMAL vs. BioMedLM

We evaluated BioMedLM on five benchmarks: BBBP and Clintox (small molecules, classification), drug–target interaction (protein–molecule, regression), antibody–antigen binding (protein–antibody, classification), and cell type annotation (multi-class classification). BioMedLM was fine-tuned using hyperparameters from the original paper. Because inputs are represented as free text, samples are large, requiring smaller batch sizes and longer training times than MAMMAL, and are limited to 1024 input tokens. For benchmarks with validation sets, the number of epochs was selected based on validation performance. For cell type annotation, which uses 5-fold cross-validation, we trained one model per fold, capping training at two days per fold. Except for small-molecule tasks, BioMedLM substantially underperformed MAMMAL (Supplementary Table S8). We hypothesize this reflects BioMedLM's training on scientific text rather than biomedical sequence data, which are central to these prediction tasks.

Table S8 Comparison of BioMedLM and MAMMAL across five benchmarks

Benchmark	Domain	Type	Metric	BioMedLM	MAMMAL	Imp.
BBBP	SM	cls	↑ AUROC	0.957	0.957	0.0 %
ClinTox	SM	cls	↑ AUROC	0.982	0.986	**0.41** %
Cell type annotation	GE	cls	↑ F1	0.509	0.758	**53.44** %
Ab-Ag binding	Protein	cls	↑ AUROC	0.567	0.928	**63.67** %
Drug target interaction	Protein+SM	reg	↓ NRMSE	1.13	0.924	**18.23** %

References

[1] Golts, A., Raboh, M., Shoshan, Y., Polaczek, S., Rabinovici-Cohen, S., Hexter, E.: Fusemedml: a framework for accelerated discovery in machine learning based biomedicine. Journal of Open Source Software **8**(81), 4943 (2023) https://doi.org/10.21105/joss.04943

[2] Suzek, B.E., Wang, Y., Huang, H., McGarvey, P.B., Wu, C.H., UniProt Consortium: UniRef clusters: a comprehensive and scalable alternative for improving sequence similarity searches. Bioinformatics **31**(6), 926–932 (2015) https://doi.org/10.1093/bioinformatics/btu739

[3] Consortium, T.U.: UniProt: the Universal Protein Knowledgebase in 2023. Nucleic Acids Research **51**(D1), 523–531 (2022) https://doi.org/10.1093/nar/gkac1052 https://academic.oup.com/nar/article-pdf/51/D1/D523/48441158/gkac1052.pdf

[4] Olsen, T.H., Boyles, F., Deane, C.M.: Observed antibody space: A diverse database of cleaned, annotated, and translated unpaired and paired antibody sequences. Protein Science **31**(1), 141–146 (2022) https://doi.org/10.1002/pro.4205 https://onlinelibrary.wiley.com/doi/pdf/10.1002/pro.4205

[5] Szklarczyk, D., Kirsch, R., Koutrouli, M., Nastou, K., Mehryary, F., Hachilif, R., Gable, A.L., Fang, T., Doncheva, N., Pyysalo, S., Bork, P., Jensen, L., von Mering, C.: The STRING database in 2023: protein–protein association networks and functional enrichment analyses for any sequenced genome of interest. Nucleic Acids Research **51**(D1), 638–646 (2022) https://doi.org/10.1093/nar/gkac1000 https://academic.oup.com/nar/article-pdf/51/D1/D638/48440966/gkac1000.pdf

[6] Biology, C.S.-C., Abdulla, S., Aevermann, B., Assis, P., Badajoz, S., Bell, S.M., Bezzi, E., Cakir, B., Chaffer, J., Chambers, S., et al.: Cz cellxgene discover: A single-cell data platform for scalable exploration, analysis and modeling of aggregated data. BioRxiv, 2023–10 (2023) https://doi.org/10.1093/nar/gkae1142

[7] Theodoris, C.V., Xiao, L., Chopra, A., Chaffin, M.D., Al Sayed, Z.R., Hill, M.C., Mantineo, H., Brydon, E.M., Zeng, Z., Liu, X.S., Ellinor, P.T.: Transfer learning enables predictions in network biology. Nature **618**(7965), 616–624 (2023) https://doi.org/10.1038/s41586-023-06139-9

[8] Kim, S., Chen, J., Cheng, T., Gindulyte, A., He, J., He, S., Li, Q., Shoemaker, B.A., Thiessen, P.A., Yu, B., Zaslavsky, L., Zhang, J., Bolton, E.E.: PubChem 2023 update. Nucleic Acids Research **51**(D1), 1373–1380 (2022) https://doi.org/10.1093/nar/gkac956 https://academic.oup.com/nar/article-pdf/51/D1/D1373/48441598/gkac956.pdf

[9] Tingle, B.I., Tang, K.G., Castanon, M., Gutierrez, J.J., Khurelbaatar, M., Dandarchuluun, C., Moroz, Y.S., Irwin, J.J.: Zinc-22-a free multi-billion-scale database of tangible compounds for ligand discovery. Journal of Chemical Information and Modeling **63**(4), 1166–1176 (2023) https://doi.org/10.1021/acs.jcim.2c01253 https://doi.org/10.1021/acs.jcim.2c01253. PMID: 36790087

[10] Raffel, C., Shazeer, N., Roberts, A., Lee, K., Narang, S., Matena, M., Zhou, Y., Li, W., Liu, P.J.: Exploring the limits of transfer learning with a unified text-to-text transformer. Journal of Machine Learning Research **21**(140), 1–67 (2020)

[11] Kong, X., Huang, W., Liu, Y.: End-to-end full-atom antibody design. arXiv preprint arXiv:2302.00203 (2023) https://doi.org/10.48550/arXiv.2302.00203
